# Functional Conservation and Divergence of Four Ginger *AP1*/*AGL9* MADS–Box Genes Revealed by Analysis of Their Expression and Protein–Protein Interaction, and Ectopic Expression of *AhFUL* Gene in *Arabidopsis*

**DOI:** 10.1371/journal.pone.0114134

**Published:** 2014-12-02

**Authors:** Xiumei Li, Tian Fan, Juanjuan Song, Wei Sun, Kuaifei Xia, Jingping Liao, Mingyong Zhang

**Affiliations:** 1 Key Laboratory of Plant Resources Conservation and Sustainable Utilization, South China Botanical Garden, Chinese Academy of Sciences, Guangzhou 510650, China; 2 University of Chinese Academy of Sciences, Beijing 100049, China; 3 Institute of Chinese Materia Medica, Chinese Academy of Chinese Medical Science, Beijing, 100700, China; 4 Key Laboratory of South China Agricultural Plant Molecular Analysis and Genetic Improvement, South China Botanical Garden, Chinese Academy of Sciences, Guangzhou 510650, China; University of Naples Federico II, Italy

## Abstract

*Alpinia* genus are known generally as ginger–lilies for showy flowers in the ginger family, Zingiberaceae, and their floral morphology diverges from typical monocotyledon flowers. However, little is known about the functions of ginger MADS–box genes in floral identity. In this study, four *AP1/AGL9* MADS–box genes were cloned from *Alpinia hainanensis*, and protein–protein interactions (PPIs) and roles of the four genes in floral homeotic conversion and in floral evolution are surveyed for the first time. *AhFUL* is clustered to the *AP1*lineage, *AhSEP4* and *AhSEP3b* to the *SEP* lineage, and *AhAGL6–like* to the *AGL6* lineage. The four genes showed conserved and divergent expression patterns, and their encoded proteins were localized in the nucleus. Seven combinations of PPI (AhFUL–AhSEP4, AhFUL–AhAGL6–like, AhFUL–AhSEP3b, AhSEP4–AhAGL6–like, AhSEP4–AhSEP3b, AhAGL6–like–AhSEP3b, and AhSEP3b–AhSEP3b) were detected, and the PPI patterns in the *AP1*/*AGL9* lineage revealed that five of the 10 possible combinations are conserved and three are variable, while conclusions cannot yet be made regarding the other two. Ectopic expression of *AhFUL* in *Arabidopsis thaliana* led to early flowering and floral organ homeotic conversion to sepal–like or leaf–like. Therefore, we conclude that the four *A. hainanensis AP1/AGL9* genes show functional conservation and divergence in the floral identity from other MADS–box genes.

## Introduction

Angiosperm flowers are quite diverse in their morphology. Orthologous genes of different taxa can display divergent functions, which may provide the genetic basis for the floral diversification among flowering plants [1]–[4]. Intensive molecular and genetic analyses in several eudicot species, particularly the four–whorled flower *Arabidopsis thaliana* and snapdragon (*Antirrhinum majus*), established the ABC model [5]. The identity of the four whorl floral organs (sepals, petals, stamens, and carpels) in eudicots is specified by floral homeotic MADS–box genes of class A, A+B, B+C, and C, respectively. However, the ABC model is not sufficient to explain all angiosperm flowers; for example, Liliaceae flowers were exemplified by van Tunen et al. [6] as the basis of a modified ABC model. The ABC model is still being modified to account for additional data from different plants [7], particularly data on the formation of heterodimers or homodimers [8]–[11], together with successive findings of E class and ovule–specific D class proteins in petunia, tomato, and *Arabidopsis* [12]–[16]. As a result, the classic ABC model was expanded to the “ABCDE model”, in which combinations of the A/B/C/D/E class genes specify the identity of each organ and control floral meristem determinacy [17], [18]. According to this model, the identity of the different floral organs is determined by four combinations of floral homeotic MADS–box proteins [17], [18].

The temporal and spatial expression patterns of the A/B/C/D/E class genes and the complicated protein–protein interaction (PPI) patterns are the molecular basis for flower development [19]. Most A, B, C, D, and E class genes belong to a conserved sequence MIKC^C^–type MADS–box gene family, except the *APETALA2* (*AP2*) gene, which belongs to another gene family of DNA–binding proteins [20]–[23]. In *planta*, the history of the MADS–box gene family is characterized by duplication events and subsequent divergence [1], [24]–[26]. The ABC and ABCDE models provide an explicit reference to the floral morphology differences correlated with genes divergence in other taxa [2], [27], [28].

In plants, the *APETALA1* (*AP1*) genes are grouped with the *SEPALLATA* (*SEP*) genes and *AGAMOUS LIKE6* (*AGL6*) genes in a monophyletic lineage named as the *AP1*/*AGL9* (*AP1/SEP/AGL6* lineage) [26], [29], [30]. The *AP1* lineage consists of two clades (eu*FUL* and eu*AP1*) in core eudicots, and *FRUITFULL* (*FUL*)–like genes in monocot, magnoliid, and basal eudicots [24], [31]. The *AP1* lineage genes have been implicated in the specification of floral meristem identity, as well as sepal and petal identity [32]–[35]. Three partially redundant genes (*AP1*, *FUL*, and *CAULIFLOWER* [*CAL*]) share a function in floral meristem specification, which was characterized by comparing single, double, and triple mutants in *Arabidopsis* [36], [37]. Furthermore, *AP1* has a unique role for specification of sepal and petal identity, while *FUL* acts to promote the flowering pathway and regulate fruit development [38].

The E class genes were defined through the discovery of *SEP*–like genes, which are required for the specification of sepals, petals, stamens, and carpels [15]. The *SEP* subfamily of the MADS–box genes underwent a duplication, resulting in two clades, the *AGL9* group and the *AGL2/3/4* group (also named *LOFSEP* and *SEP3*) [39], [40]. The best known the E class genes are *SEP1* (*AGL2*), *SEP2* (*AGL4*), *SEP3* (*AGL9*), and *SEP4* (*AGL3*) of *Arabidopsis* [18], [39]. The expression patterns in the *AGL9* and *AGL2/3/4* groups are slightly different; *SEP1* and *SEP2* are expressed in all four whorls and *SEP4* is expressed more highly in carpel primordium than in the sepals, whereas *SEP3* is only expressed in the inner three whorls of the *Arabidopsis* flower [15], [41], and they have been thought as functionally redundant genes [15], [18]. *SEP* genes act as integrating co–regulators with other MADS–box genes in floral organ specification [42]. The E class genes appeared to be essential for organ identity specification in all floral whorls; analyses at the protein level revealed that the E proteins act as “bridging molecules” and mediate the physical interactions between A and B, B and C, and C and the ovule–specific D class proteins [43]–[48].

*AGL6* clade genes comprise a sister clade of the *SEP* genes and may share E class functions; both clades are recovered as monophyletic [39], [49]. The *AP1/FUL* and the *SEP* lineages derived from a single ancestral tandem duplication in a common ancestor of extant seed plants through a combination of genome synteny and phylogenetic reconstructions [50]. The *AGL6* lineage together with the *AP1/FUL* lineage and *SEP* lineage form a highly supported clade [30], [39], [51], but *AGL6* is the only remaining clade in gymnosperms.

The floral MIKC^C^–type MADS–box genes encode transcription factors that regulate the expression of their downstream genes by forming various dimers and complexes [11], [46], [52]–[54]. According to the “quartet model”, the formation of various dimers is a prerequisite for the formation of higher–level protein complexes [17], [18]. The evolution of MADS–box genes as well as interactions among proteins encoded by these genes will help to understand the mechanisms that underlie morphological innovations in the flower [55], [56]. Comparing the network in different species showed that conservativeness and variability co–exist in composition, organization, and structure of the complex interactions among proteins encoded by these genes [28], [57], [58]. Research into PPIs of the floral MADS–box genes has been concentrated in the higher eudicots, and in monocots mainly focused on Poaceae and Orchidaceae.

The ginger family (Zingiberaceae) is one of eight families of monocotyledons that make up the order Zingiberales [59]–[61]. Many species are important ornamental plants with high agronomical values, and are used as spices or medicinal plants. Their floral morphology shows divergence from typical monocotyledon flowers. A “typical” monocotyledon flower is trimerous and contains six stamens arranged in two whorls. In contrast, among flowers of Zingiberaceae, the sepals are either united into a synsepalous calyx; the petals are united with all other floral organs, including the androecium and style, into a floral tube of varying extent and complexity; the number of stamens that produce pollen is reduced to one [59], [60]. *Alpinia hainanensis*, a member of the *Alpinia* genus in the ginger family, is characterized by flexistyly, in which the style moves with the timing of anther dehiscence during flowering [62], and bears one large showy labellum and with two small subulate lateral staminodes. It has been hypothesized that the evolution and function diversification of the MADS–box transcription factors contributed to the variety of floral morphology in the Zingiberales [63]. Hence, do orthologs of the ginger MADS–box genes have similar functions in controlling the individual plant ontogeny, especially in floral development? Little is known regarding the role of the MADS–box genes in regulating flower formation and flower initiation in the gingers. Here, three putative *AP1*– and *SEP*– lineage genes and one *AGL6*–like gene in the *AP1*/*AGL9* group from the ornamental ginger *A.hainanensis* (Zingiberaceae) were cloned; understanding the protein interaction pattern and the floral organ identity may shed light on the potential roles of MADS–domain proteins in the ginger floral development and evolution.

## Materials and Methods

### Plant material

Floral buds, young flowers, and floral organs of *A. hainanensis* at various developmental stages were collected from the ginger garden in the South China Botanical Garden, Chinese Academy of Sciences.

Seeds for *Arabidopsis* were sterilized and placed on agar plates containing 1/2× Murashige and Skoog (MS) medium [64] at 4°C for 2 days. The seedlings were then grown in growth chambers under long day conditions (16–h light/8–h dark) at 22°C for 10 days before being transplanted to soil.

### RNA extraction, gene isolation, and quantitative RT–PCR

Total RNA of floral organs and of different stages was extracted from multiple flowers using RNA reagent (TIANDZ, Beijing, China). 5′ and 3′ rapid amplification of cDNA ends (RACE) were used to isolate the full–length cDNA of *AhSEP3b* genes with degenerate primers, following the SMARTer RACE cDNA Amplification Kit User Manual of Clontech (Takara Bio Company). The other three genes (*AhFUL*, *AhSEP4*, and *AhAGL6–like*) were isolated as previously described [65]. Quantitative RT–PCR (qRT–PCR) was performed with gene–specific primers, using the 18S primer as an internal control. The relative expression of the four genes was normalized to the expression level of rRNA*18S*with biological repeats in triplicate. qRT–PCR was performed on an ABI 7500 Sequence Detection System (Applied Biosystems, Foster City, CA, USA) using a SYBR Premix Ex Taq II kit (Takara, Japan) following the manual's recommendations. The comparative Ct method was used to determine the relative expression level. The results were analyzed with ABI 7500 Software v2.0.1. All primers used in this study are listed in Table S1.

### In situ hybridization

The inflorescences of *A. hainanensis* were fixed in 4% paraformaldehyde at 4°C overnight, dehydrated, and then, embedded in paraffin. Longitudinal sections (8–µm–thick) cut from the embedded tissue were transferred onto poly–D–Lys coated slides. The C–terminus and 3′untranslated regions of two cDNA clones were amplified by PCR and introduced into the pGEM–T Easy vector. Linear DNA were used as templates to synthesize digoxygenin–labeled antisense and sense RNA probes. And the probes were applied to sections at a final concentration of 10 ng/mL.

### Sequence alignment and phylogenetic analyses

Homologous analyses of the predicted protein sequences were performed by the BLASTX program in the National Center for Biotechnology Information (NCBI). The structure of protein sequences were predicted in predict protein (https://www.predictprotein.org). The protein sequences (Table S2) were aligned using ClustalX 1.83 [66], and the alignment was manually adjusted in Bioedit V.7.0.9 [67]. A phylogenetic tree was constructed with MEGA 5 using the neighbor–joining method [68]. Numbers on the tree nodes are the bootstrap values from 1000 replicates.

### Subcellular localization of green fluorescent protein fusion proteins

Subcellular localization prediction was carried out using WoLF PSORT Prediction (http://wolfpsort.org). The open reading frames of *AhFUL*, *AhAGL6–like*, *AhSEP4*, and *AhSEP3b* without the stop codon were inserted into pBI221–GFP vectors (Clontech, CA, USA), resulting in constructs containing the green fluorescent protein (GFP) fused at the C–terminus of the four proteins. The four constructs were transferred into *Arabidopsis* mesophyll protoplasts using PEG–mediated transformation [69], [70]. Protoplasts harboring the empty pBI221–GFP vector (*35S:GFP*) were used as a control. The GFP signals were monitored under a confocal spectral microscope (Leica CP SP2, Germany).

### Bimolecular fluorescence complementation

The four vectors in the bimolecular fluorescence complementation (BiFC) assay [pSAT1–cEYFP–C1(B), pSAT1–nEYFPC1, pSAT1(A)–cEYFP–N1, and pSAT1(A)–nEYFP–N1], expressing truncated yellow fluorescent protein (YFP) driven by two cauliflower mosaic virus 35S promoters, oriented in tandem, were obtained from the Department of Biological Sciences, Purdue University (https://www.bio.purdue.edu/people/faculty/gelvin/nsf/protocols_vectors.htm).

The full–length coding sequences of *AhFUL*, *AhSEP4*, *AhAGL6–like*, and *AhSEP3b* were fused with the N–terminal fragment of YFP in the pSAT1–nEYFP–C1 and the pSAT1(A)–nEYFP–N1 vectors, while the four coding sequences were also cloned into pSAT1–cEYFP–C1(B) and pSAT1(A)–cEYFP–N1 as a fusion with the C–terminal fragment of YFP. For each pair of protein interaction partner, amino– and carboxyl– terminal fusions are can be used to test eight combinations. All empty vectors were used as negative controls, and the bZIP63–pSPYNE and bZIP63–pSPYCE vectors were used as positive controls [71]. These constructs were transiently expressed in *Arabidopsis* mesophyll protoplasts according to previously reported procedures [69], [70]. mCherry–VirD2NLS was induced in each transfection to serve as a control for successful transfection as well as for nuclear localization [72]. The transfected cells were imaged using an epifluorescence microscope (Leica) or/and a TCS SP5 Confocal Spectral Microscope Imaging System (Leica), with an argon blue laser at 488 nm, a beam splitter for excitation at 500 nm, and a spectral detector set between 515 nm and 540 nm. The combinations of BiFC have been performed at least two biological replicates, some combinations that were found to be localized in cytoplasm have been performed more than two replicates.

### Reconstruction ancestral states of PPI patterns

To trace the conservation and diversification of the PPI patterns among the MADS–box genes across angiosperms, we used character–state reconstructions in Mesquite version 2.75 (available from http://mesquiteproject.org) (Maddison WP and Maddison DR: Mesquite: a modular system for evolutionary analysis. Version 2.75. 2011). PPI data obtained in this study and from published articles (Table S3) were combined before they were integrated into new matrices (Table S4). For each matrix, only species with available interaction data were included, and topologies reflecting the phylogenetic relationships of these species were used as input trees. The phylogenetic trees were generated in Phylomatic Version 3 (http://phylodiversity.net/phylomatic) based on the APGIII [73]. For each species, one of the two states (0 for absence of interaction and 1 for presence of interaction) were assumed and mapped onto the phylogenetic trees if there was only one gene or if the two or more paralogous genes showed the same interaction behavior. Ancestral states of the PPIs at the ancestral nodes of each phylogenetic tree were traced by using likelihood and parsimony methods in the “Trace Character History” function of Mesquite. In the likelihood analyses, the Markov *k*–state one–parameter model [74], which treats all changes as equally probable, was applied because the more comprehensive asymmetrical 2–parameter Markov *k*–state model was rejected. In the parsimony analyses, the unordered model, in which all state changes are treated equally, was applied.

### Plant transformation and transgenic plants analysis

A *Kpn*I and *Sal*Ifragment containing the cDNA for *AhFUL*, and a *Sac*I and *Bam*HI fragment containing the cDNA for the *AhSEP4* gene were cloned into binary vector pCAMBIA2301 (CAMBIA) under the control of the CaMV 35S promoter. These constructs were transformed into *Arabidopsis* plants using the floral dip method [75]. Transformants were selected in medium containing 50 µg mL^−1^ kanamycin and were further verified by PCR and RT–PCR analyses.

### Scanning electron microscopy

Transgenic *Arabidopsis* plants for scanning electron microscopy (SEM) were collected and fixed using formalin–acetic acid–alcohol for 2 days and then stored in 70% alcohol. Specimens used for SEM were dehydrated in an alcohol series running up to 100%, treated with isoamylacetate, critical point dried using CO_2_, and sputter–coated with gold palladium in six 30 s bursts (JEE–420, Hitachi, Tokyo, Japan). SEM was performed with a JSM–6360LVscanning electron microscope at 15 kV (Hitachi, Tokyo, Japan), and SEM photographs were processed with Adobe Photoshop 8.0 software.

## Results

### Isolation and sequence analyses of four *A. hainanensis* MADS–box genes

**Figure 1:**
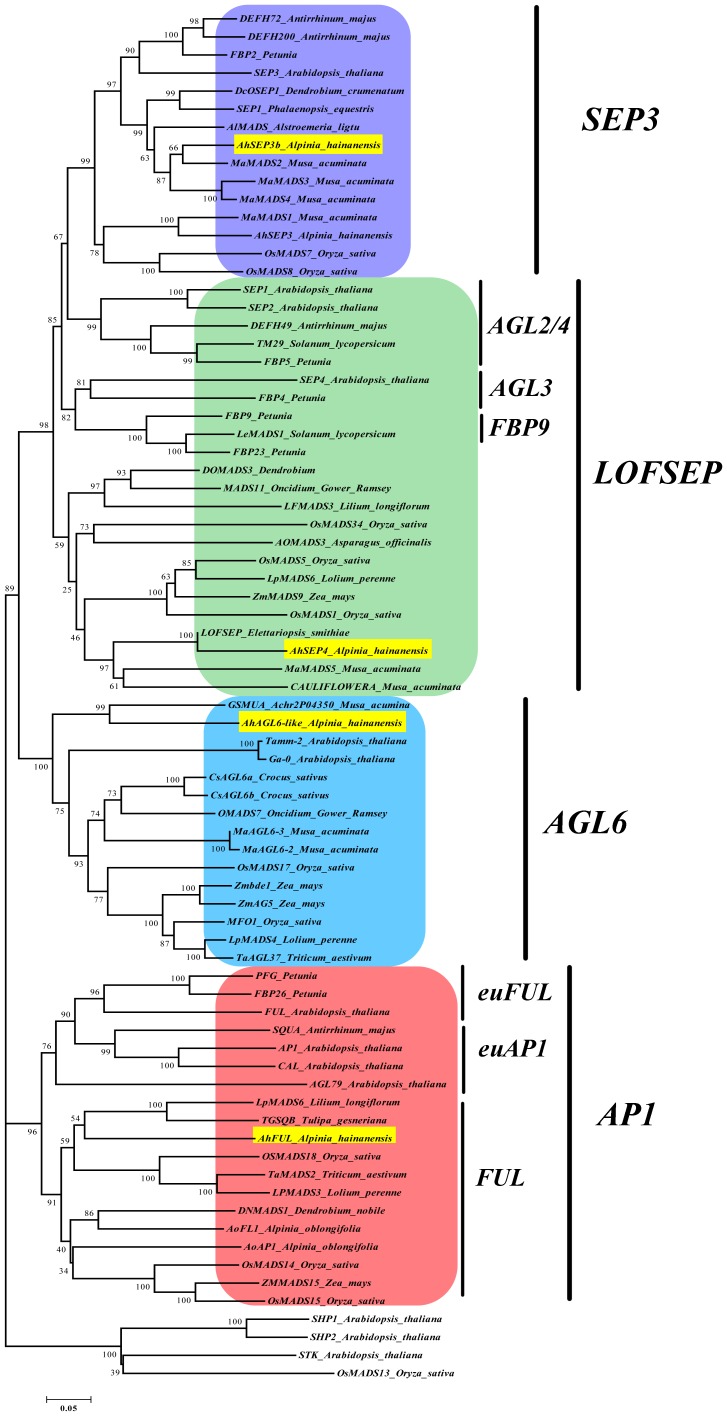
Phylogenetic tree of AP1/AGL9–like MADS–box proteins. AP1/AGL9 lineage of the MADS–box genes could be further divided into four clades (AP1/AGL6/LOFSEP/SEP3). On the basis of amino acid sequence of the full–length protein, some differences with canonical *AGL6* genes may explain the position of AhAGL6–like close to *AGL6* lineage but in the external branch of this clade. MEGA 5 software was used with the neighbor–joining method using the parameters of p–distance, complete deletion, and bootstrap (1000 replicates). The four genes from *A.hainanensis* are labeled in yellow. The term “out” indicates the outgroup (D class genes in *Arabidopsis* and *Oryza sativa*) used for the analysis. Information of these genes is listed in Table S2.

To understand the role of MADS–box gene family in the flower development of *A.hainanensis*, we isolated four genes from the developing flowers by cDNA library construction and RACE using degenerate primers (Table S1). Homologous analysis suggested that all four genes belong to the MIKC^C^–type MADS–box gene family [76] (Figure S1A, B). A phylogenetic tree (Figure 1) generated using amino acid sequences shows that One (designated *AhFUL*) belongs to the *AP1* lineage, two (designated *AhSEP4* and *AhSEP3b*) to the *SEPALLATA* lineage, and the remaining one (designated *AhAGL6–like*) is close to the *AGL6* lineage of the MADS–box gene family, which were further verified by C–terminal sequence alignment (Figure S1A). Their sequence information is listed in Table S5.

*AhFUL* mRNA was predicted to encode a protein with 244 amino acid residues, which is highly homologous (60%, 61%, and 51% identity, respectively) to *A.oblongifolia* AoFL1 [77], *Lilium longiflorum* MADS6 [78], and *Arabidopsis* FUL [38] (Figure S1). The AhFUL protein contains four typical motifs (M, I, K, and C) of the plant–specific FUL–type proteins, a FUL motif, and a variable paleoAP1 (LLPPWML) motif (Figure S1) [24], [79]. These results suggest that *AhFUL* may be a *FUL* ortholog of *A. hainanensis*.

*AhAGL6–like* mRNA was predicted to encode a protein with 240 amino acid residues, which shows 65%, 59%, and 57% identity to *Crocus sativus* CsAGL6, *Oncidium Gower Ramsey* OMADS7, and *Oryza sativa* MFO1 of the *AGL6* lineage [19], [80] (Figure S1). However, the C–terminal regions of the AhAGL6–like protein are different from most AGL6–like proteins in the two conservative motifs (AGL6–I and AGL6–II) [19]. The AGL6–I motif (DCEPTLQIGY) is substituted by the sequence (ECQPTPQIRY) in AhAGL6–like, and three amino acids (NFM) are absent from the AGL6–II motif (ENNFMLGWVL) in AhAGL6–like (Figure S1). These data indicate that *AhAGL6–like* may be an*AGL6*–like gene of the MADS–box gene family with a different function.

The predicted proteins AhSEP4 and AhSEP3b (to distinguish the AhSEP3 isolated before [81]) contain 215 and 242 amino acid residues, respectively, and they are grouped into the *SEP* subfamily (Figure 1). Both of them contain the typical SEP–I and SEP–II motifs (Figure S1) in the C–termini, like most SEP proteins [39]. AhSEP4 showed 53% identity to *Oncidium Gower Ramsey* MADS11 [80], and AhSEP3b showed 89% identity to *Musa acuminate* MADS2 [82]. These data suggest that *AhSEP3b* and *AhSEP4* may be *SEP* genes, and close to the *SEP3* and *SEP1*/*2*/*4* genes, respectively.

### Expression patterns of four MADS–box genes of *A. hainanensis*

**Figure 2:**
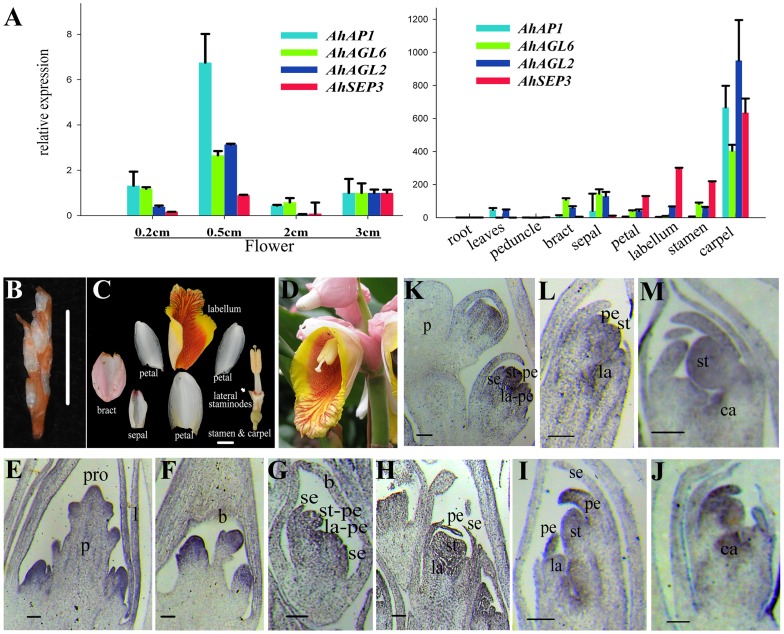
Flower morphology of *A. hainanensis*, and expression analyses of *AhFUL, AhSEP3b, AhAGL6–like*, and*AhSEP4*. (A) Expression analyses of four genes by quantitative RT–PCR in different organs of the 2 cm length flowers (right), and in flowers at different developmental stages (left). The relative expression of the four genes was normalized to the expression level of rRNA *18S* with biological repeats in triplicate. (B)An early developing *A. hainanensis* in florescence with flower buds. (C) Anatomical structure of an *A. hainanensis* flower, consisting of one bract, one tubular sepal, three petals, one labellum with two lateral staminodes, one stamen and carpel. (D) A mature flower in the *A. hainanensis* inflorescence. (E–M) *In situ* hybridization patterns of *AhFUL* (E–J) and *AhAGL6–like* (K–M) transcripts in longitudinal sections of *A. hainanensis* flowers. Leave (l), bract (b), sepal (se), petal (pe), common primordium of stamens and petals (st–pe), common primordium of the labellum and petals (la–pe), stamen (st), labellum (la), carpel (ca). Bar  = 1 cm in (B) and (C), 100 µm in (E–M).

We investigated the expression patterns of the four genes by using the RT–PCR and/or RNA *in situ* hybridization techniques, respectively (Figure 2). Quantitative RT–PCR was used to evaluate the expression difference of the four ginger *AP1/AGL9* genes in the developing flower at 0.2, 0.5, 2, and 3 cm length stages (Figure 2A left), and different floral organs of the 2 cm flowers (Figure 2A right). The transcripts of *AhFUL* were first detected in the apex of the floral primordium and bract (Figure 2E), whereas relative weaker signal in inflorescence bract and leaves (Figure 2E, F). Then, the transcripts were accumulated in common primordia of stamen–petal and labellum–petal (Figure 2G, H). The *AhFUL* was expressed in the petals, labellum and stamen after the petals differentiated from the common primordia, with the *AhFUL* transcripts mainly being in the regions where cells were actively dividing (Fig. 2I, J). In the mature floral organs, the transcripts of *AhFUL* were maintained in the sepals but not in the petals, labellum and stamen (Figure 2A right). For *AhAGL6–like*, the signals were transferred from common primordia of stamen–petal and labellum–petal to stamen and carpel but weak in labellum with the differentiation of floral organs (Figure 2K–M). *AhAGL6–like* was expressed in all mature six floral organs but not in the vegetative organs, while its expression remained weak in labellum. *AhSEP4* transcript was detected in leaves of the vegetative organs and the six floral organs, while *AhSEP3b* expression was restricted to four floral organs (petal, labellum, stamen, and carpel). The *AP1*/*AGL9* genes in *A. hainanensis* had broader expression regions than their counterparts in core eudicots.

### PPI patterns among four MADS–box genes of *A. hainanensis*

**Figure 3:**
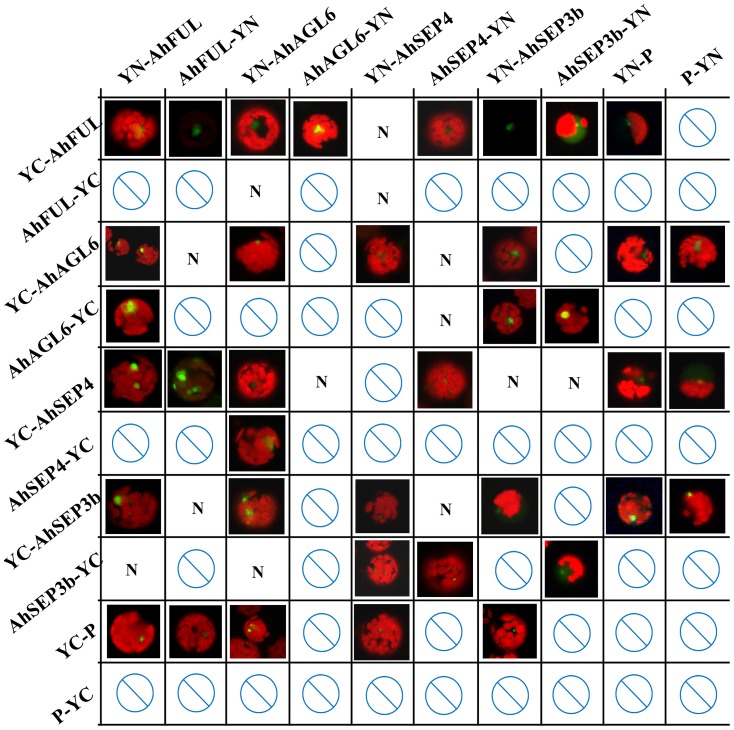
Physical protein–protein interaction of AhFUL, AhSEP4, AhSEP3b, and AhAGL6–like proteins by bimolecular fluorescence complementation (BiFC). Results of BiFC analyses in *Arabidopsis* mesophyll protoplast transient expression system showed that AhFUL–AhSEP4, AhFUL–AhAGL6–like, AhFUL–AhSEP3b, AhSEP4–AhAGL6–like, AhSEP4–AhSEP3b, AhAGL6–like–AhSEP3b, and AhSEP3b–AhSEP3b could form dimers. The full–length coding sequences of AhFUL, AhSEP4, AhAGL6–like, and AhSEP3b were fused with the N–terminal fragment of YFP in the pSAT1–nEYFP–C1 (YN–P) and the pSAT1 (A)–nEYFP–N1 (P–YN) vectors, while the four coding sequences were also cloned into pSAT1–cEYFP–C1(B) (YC–P) and pSAT1(A)–cEYFP–N1 (P–YC) as a fusion with the C–terminal fragment of YFP. Amino– and carboxyl– terminal fusions are represented by YC–P and YN–P, P–YC and P–YN respectively. All empty vectors were used as negative controls. mCherry–VirD2NLS was induced in each transfection to serve as a control for successful transfection as well as for nuclear localization. Symbols of cross–lined circles: negative interactions; N: no test, because fluorescence can be detected in the protein with empty vector.

In addition to comparing genes from different species by sequence alignment and expression pattern, the analysis of conserved PPIs can be used to compare putative functional homologs, as already demonstrated for MADS–box factors from other species. To investigate whether PPIs exist among the four ginger *AP1*/*AGL9* genes, BiFC was performed (Figure 3) in *Arabidopsis* mesophyll protoplasts transient expression systems [83]. The results showed that AhFUL, AhSEP4 and AhAGL6–like cannot form homodimers, while the heterodimers were able to form between the two of four genes and the YFP signals were detected in the nucleus (Figure 3, Figure S2). PPIs of AhSEP3b–AhAGL6–like, AhSEP3b–AhSEP3b, AhFUL–AhFUL and AhAGL6–like–AhAGL6–like, which may divergent with other species, were further verified by yeast two-hybrid (Y2H) (Figure S3). Some combinations were found to be localized in cytoplasm after 8 h incubation at 25°C (Figure S4A); however, they were localized in the nucleus when the incubation time increased to 16–18 h (Figure S4B). This indicates that sufficient time is required for translocation of the combined proteins from the cytoplasm to the nucleus. Since fluorescent cells were observed in the leaf protoplasts in some negative controls, these results of PPI pairs were excluded. In addition, a necessary condition for PPI between two different proteins is that they should co–exist in the same tissue at the same time. The four genes are co–expressed in floral organs; their PPIs are possible but do not necessarily occur. How these dimers play cooperative roles in floral transition and organ development should be investigated further.

### Four ginger MADS–box proteins are localized in the nucleus

**Figure 4:**
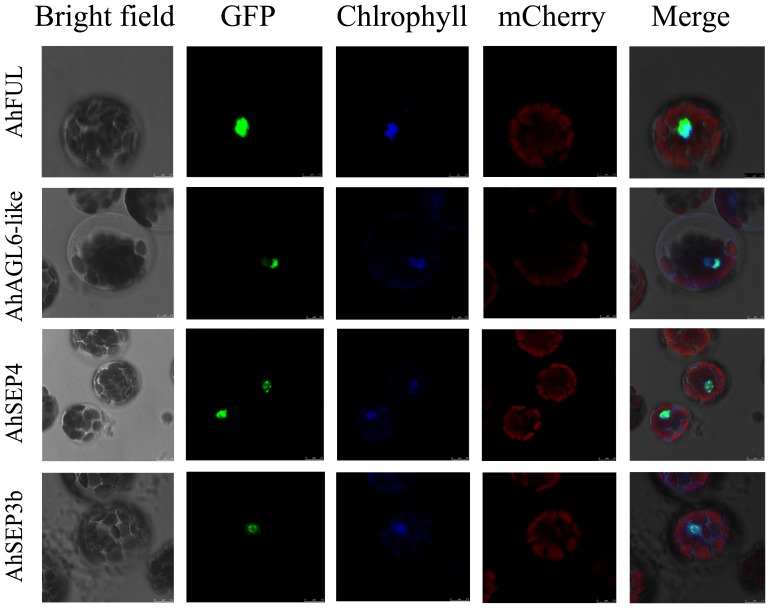
Subcellular localization of four ginger MADS–box proteins. AhFUL–GFP, AhAGL6–like–GFP, AhSEP4–GFP, and AhSEP3b–GFP fusion proteins under control of the CaMV*35S* promoter were transiently expressed in *Arabidopsis* mesophyll protoplasts. Images were taken in the dark field for green fluorescence, while the outline of the cell and the combination were photographed in a bright field. mCherry–VirD2NLS was induced in each transfection to serve as a control for successful transfection as well as for nuclear localization. The length of the bar is indicated in the photographs.

The MADS–box proteins contain the conserved DNA–binding domain as transcription factors and are localized in the nucleus. Most MADS–box proteins contain nuclear localization signal sequences (K–K/R–x–K/R) in the N–terminal–located MADS domain; furthermore, this domain should be present in both interacting partners for transport of the dimer to the nucleus [84]. Therefore, it is interesting to investigate the subcellular localization of the four ginger *AP1*/*AGL9* proteins in plant cells. After we found the dimers (AhAGL6–like–AhSEP3b, AhSEP3b–AhSEP3b) (Figure 3) were localized in the nucleus, their GFP fusion proteins were analyzed. These fusion proteins were transiently expressed in *Arabidopsis* protoplasts, and cells were imaged with a laser scan confocal microscope. The fluorescence signals of all these fusions (*AhFUL*–GFP, *AhAGL6–like*–GFP, *AhSEP3b*–GFP, and *AhSEP4*–GFP) were localized exclusively to the nucleus (Figure 4), while the fluorescence of GFP alone was observed throughout the cells (Figure S5). These assays demonstrate that all the four ginger MADS–box proteins were targeted into the nucleus.

### Evolutionary reconstruction of PPI patterns among *AP1/AGL9*

**Figure 5:**
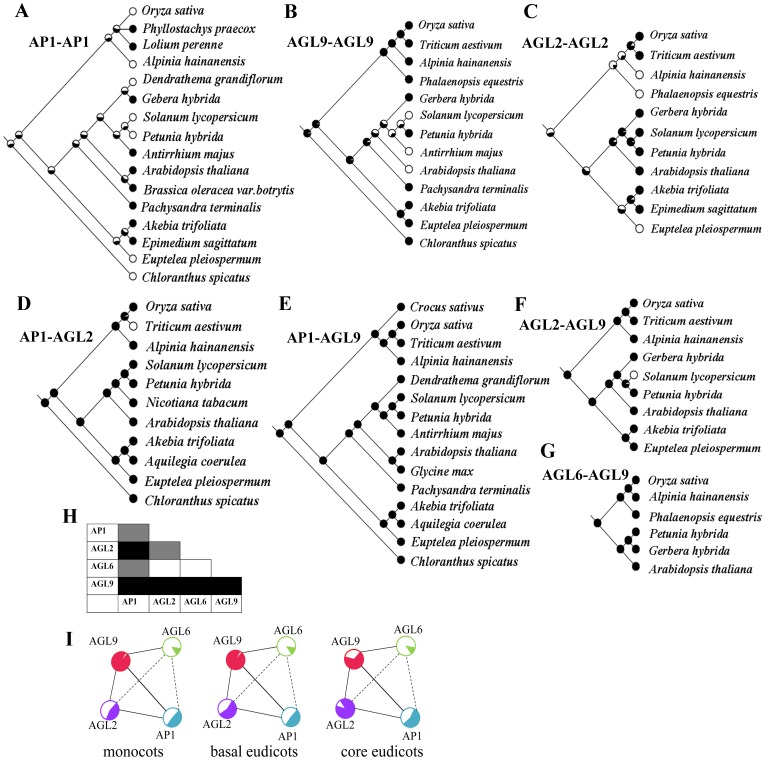
Evolutionary reconstruction of protein–protein interaction (PPI) pattern between *AP1*/*AGL9* lineage proteins. Ancestral character–state reconstructions of PPIs between proteins of the AP1, AGL2, AGL6, and AGL9 lineage members. Filled and open circles in (A–G) indicate presence and absence of interactions, respectively, with the probability of the interaction in ancestral taxa indicated at each interior node. Among the 10protein interaction combinations (H), five had conserved (in black) and three had variable (in grey) PPIs. In the remaining 2 cases, no interaction was observed or accumulated data so far is unavailable. Information on the proteins is listed in Table S3. (I) Evolutionary historical model of the PPI network formed by AP1/AGL9 proteins, filled and open circles stand for the AP1 (blue), AGL2 (purple), AGL9 (red), and AGL6 (green) lineage members that can and cannot form homodimers.

To trace back the evolution of the regulatory network for floral development, we reconstructed the intra– and inter–lineage of the interaction patterns about *AP1*, *AGL2/3/4* (henceforth, *AGL2* for short), *AGL6*, and *AGL9* lineages from this study and published interaction data (Figure 5A–C). Study of AP1 homodimersin intra–lineage demonstrates more flexibility than that AGL2 and AGL9 homodimers. The members of AP1 that could form homodimers varied from species to species (Figure 5A). Homodimer formation in the *AGL9* lineage mainly occurred in the monocots and basal eudicots; the capability to form homodimers seems to have been gradually lost in the core eudicots (Figure 5B). Homodimer formation of the *AGL2* lineage is more or less random (Figure 5C). Although fewer data on interactions in the *AGL6* lineage are available, no homodimer formation was found in the *AGL6* lineage, including our results in *A. hainanensis*, *Petunia hybrid* [85], *Gerbera hybrid* [86], and *A. thaliana* [87]. The only homodimer formed was found in *Epimedium sagittatum* [88].

**Figure 6:**
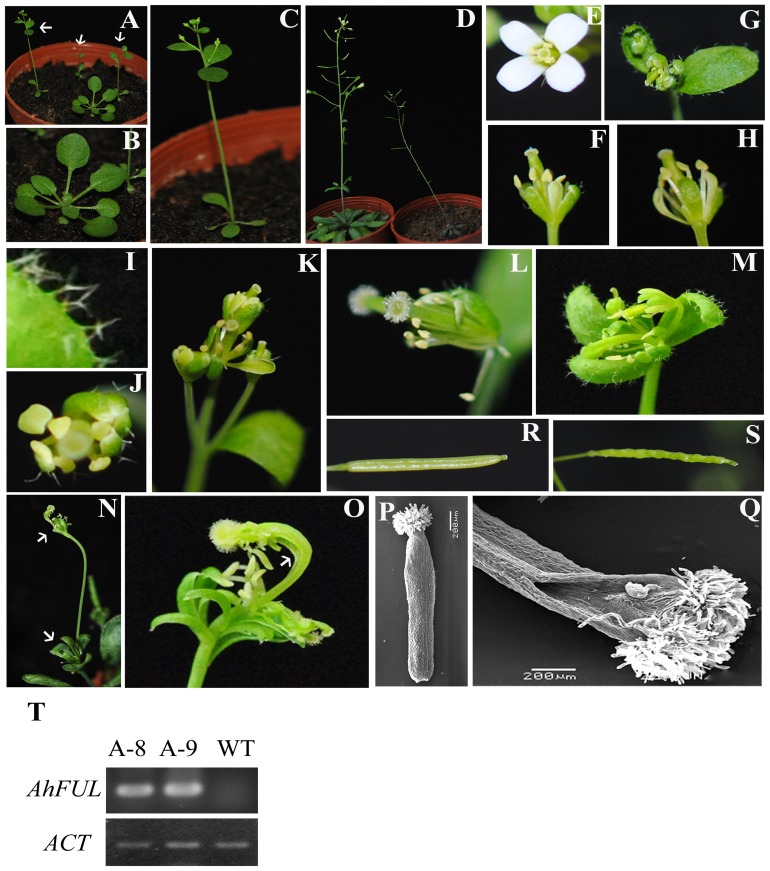
Floral organ homeotic conversion in the transgenic *Arabidopsis* plants by ectopic expression of *AhFUL*. (A) Early flowering and fewer rosettes of the 21–day–old *35S*::*AhFUL* transgenic *Arabidopsis* plant (white arrow bars) compared with the wild–type plant, when grown under long day photoperiod condition (16 h light/8 h dark). (B) The wild–type plant with round rosette leaves. (C) The *35S::AhFUL* transgenic *Arabidopsis* plants with four small curled rosettes. (D) The *35S::AhFUL* transgenic *Arabidopsis* plants were short and weak (right). (E) The wild–type flower. (F–O) Floral organ phenotypic analysis of the transgenic *Arabidopsis* ectopically expressing *AhFUL*, displaying mild (F, G, H), intermediate (I, J, K), and severe (L, M, N, O) abnormal phenotypes. White arrow bars indicate an extra flower raised from the axils of another flower. (P) Style of the wild–type plant. (Q) Style of the *35S::AhFUL* transgenic plant. (R) Siliques of the wild–type.(S) Siliques of the *35S::AhFUL* transgenic *Arabidopsis*. (T) Detection of *AhFUL* expression in transgenic *Arabidopsis* plants. Total RNA isolated from one wild–type *Arabidopsis* plant and two different 21–day–old *35S::AhFUL* transgenic plants was used as templates.

The inter–lineage analyses showed that four lineage pairs (AP1–AGL2, AP1–AGL9, AGL2–AGL9, and AGL6–AGL9) could consistently form heterodimers (Figure 6D–G). Few data regarding PPIs between *AP1* and *AGL6* lineage proteins or between *AGL6* and *AGL2* lineage proteins are available. Taken together, among the 10 PPI combinations (Figure 5H), five are relatively conservative (AP1–AGL2, AP1–AGL9, AGL2–AGL9, AGL9–AGL9 and AGL6–AGL9 in black) and another three (AP1–AP1, AP1–AGL6 and AGL2–AGL2) are variable (in grey) in PPI pattern. In the remaining 2 cases (AGL2–AGL6 and AGL6–AGL6), present information is not sufficient to draw conclusions.

### Ectopic expression of *AhFUL* causes early flowering and homeotic conversion in transgenic *Arabidopsis*

To investigate the functions of *AhFUL* and *AhSEP4* in floral organ identity and development, their cDNA driven by the CaMV*35S* promoter [89] were transformed into *Arabidopsis* plants. Twenty independent *35S::AhFUL* transgenic *Arabidopsis* plants were obtained, and 11 transgenic lines showed novel phenotypes (Figure 6). Like most *AP1/FUL*–like genes, ectopic expression of *AhFUL* in *Arabidopsis* also led to flowering early under the long photoperiod condition (16 h light/8 h dark) (Figure 6A), and caused short and weak plants (Figure 6A), compared with wild–type plants (Figure 6D). At the vegetative stage, all the transgenic *Arabidopsis* plants with *35S*::*AhFUL* produced only two to four small curled rosette leaves (Figure 6C), compared with the wild–type plant with round rosette leaves (Figure 6B).

However, abnormal floral phenotypes from mild to severe were observed among the 11 transgenic *Arabidopsis* lines with *35S*::*AhFUL* (Figure 6F–O). Two transgenic lines showed the mildest abnormal phenotypes, including size–reduction of the sepals and the petals, and the pistil protruding from the flower due to the pistil not being enclosed by the perianth (Figure 6F–H). Six transgenic lines displayed intermediate floral phenotypes, including that the first whorl developed into leaf–like organs bearing stellate trichomes, and the second whorl developed into sepal–like organs displaying light green (Figure 6I–K); while the wild–type sepals have simple, unbranched trichomes and petals are white. The most severe phenotypes of the floral homeotic conversion (3 lines) showed mosaic floral organs, whose carpelloid organs did not roll up completely to form the style as in the wild–type, while the abnormal pistil still bore ovules (Figure 6L–O, Q). The ectopic expression of *AhFUL* severely affected flower development, resulting in loss of the regular pattern of the whorl arrangement, and extra flowers raised from the axils of other flowers (Figure 6N). In addition, all six independent *35S::AhSEP4* transgenic *Arabidopsis* plants did not show floral homeotic conversion and morphologic phenotypes (data not shown). To explore if the phenotype changes in the *35S*::*AhFUL* transgenic plants correlated with *AhFUL* ectopic expression, RT–PCR analysis was performed. High expression level of *AhFUL* was in the transgenic *Arabidopsis* plant with severely abnormal flowers (Figure 6T), which indicated that the alteration of phenotypes in the*35S*::*AhFUL* transgenic *Arabidopsis* was caused by the ectopic expression of the *AhFUL* gene, and the *AhFUL* gene is involved in floral organ identity.

**Figure 7:**
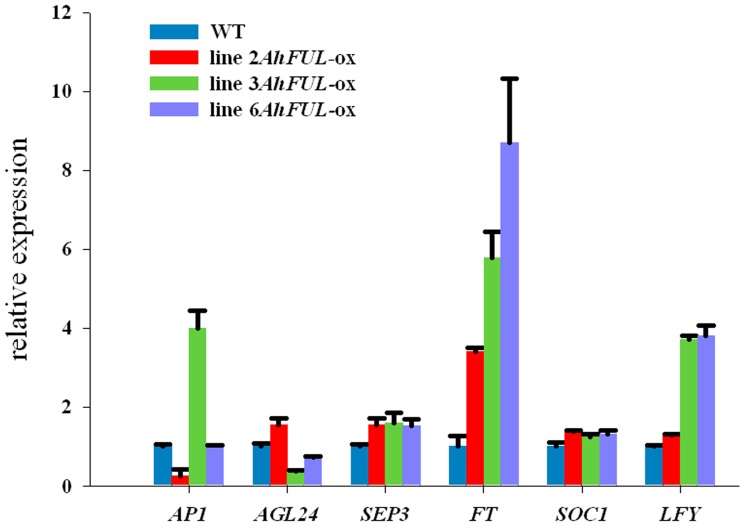
Endogenous flowering–related gene expression changes in the three *35S::AhFUL* transgenic *Arabidopsis* lines, compared with the wild–type (WT). Data represent the mean±SE from three replicate. *AP1* (NM_105581), *AGL24* (AF005158), *SEP3*, (NM_180622), *LFY* (NM_125579), *FT* (AB027505), and *SOC1* (NM_130128).

To further understand the early flowering mechanisms of the *AhFUL* transgenic *Arabidopsis*, the expression of some endogenous flowering–related genes was analyzed. Compared with the wild–type, the *LEAFY* (*LFY*) and *FLOWERING LOCUS T* (*FT*) expression in the *35S*::*AhFUL* transgenic *Arabidopsis* plants at bolting stage were up–regulated, whereas the expression of four other genes [*AGAMOUS–LIKE 24* (*AGL24*), *SUPPRESSOR OF OVEREXPRESSION OF CONSTANS1* (*SOC1*), *SEP3*, and *AP1*] did not show obvious change (Figure 7).

## Discussion

### Four *A.hainanensis AP1/AGL9* lineage MADS–box genes evolution and function diversification

Sequences evolution of proteins may be reflected by phylogenetic structure and the evolution trend in specific sites are classified in different lineages. Homologous analyses suggested that four genes (*AhFUL*, *AhAGL6–like*, *AhSEP4*, and *AhSEP3b*) are clustered into *AP1*/*FUL*, *AGL6*, *SEP3* and *SEP1*/*2*/*4* lineage, respectively (Figure 1). Sequence structures that have similar features are likely to share a relatively closer evolutionary relationship, especially if the features appear in a non–conserved region. Several conserved motifs in highly variable C–terminal regions of the MADS–box gene family [90] are also found in four *A.hainanensis AP1*/*AGL9* genes (Figure S1, Table S5), such as the SEP–I and SEP–II motifs [39], the AGL6–I and AGL6–II motifs [19], and the paleoAP1 motif in the *AP1/FUL*–like genes of monocots [24], [90], [91]. However, prior to our study, apart from *AoFL1* and *AoFL2* [92] there were no other genes available for these *AP1*–like genes from ginger. This suggests that two recent duplication event has given rise to at least three copies in the *AP1* lineage across the ginger family. Likewise, two copies of *SEP3*–like genes in *A. hainanensis* indicated that lineage–specific duplication events occurred in the diversification of the Zingiberales, resulting in two *ZinSEP3* and two *ZinLOFSEP* copies [49]. The finding of only one copy of *AGL6* in Zingiberales is consistent with other non–grass monocot lineages [49], [93].

*FUL*–like genes from basal angiosperms and basal eudicots, as well as members of the core eudicot–specific *euFUL* and *AGL79*–like genes, are generally expressed in both vegetative and reproductive organs; in contrast, the *euAP1* members were never detected in roots or leaves [79], [80], [91], [94]–[96]. The *AP1* lineage initially had broad functions in transition to flowering and floral organ identity, the functions may have been later partitioned between different paralogous gene copies following duplication and neofunctionalization in the core eudicots [79], [88]. The broad expression pattern of *AhFUL* implies that it may possess putative ancestral function in the *AP1* lineage. Nevertheless, the expression pattern is divergent among *AhFUL*, *OsMADS18* from *Oryza,* and *FUL* from *Arabidopsis* (Table S6); the expression region in *AhFUL* and *OsMADS18* is broader than in *Arabidopsis*. *AhFUL* was expressed not only in the floral organs, but also in the vegetative organs (Figure 2), which indicates that the role of *AhFUL* is not restricted to floral development; it is also involved in leaf development. This may be supported by the abnormal leaves observed on the *35S::AhFUL* transgenic *Arabidopsis* (Figure 6). Expression in the formed carpel of mature flowers distinguished *AhFUL* from *AoFL1* and *AoFL2* in *Alpinia oblongifolia*, this suggested that the *AP1/FUL*–like genes have evolved divergent expression patterns in response to diversified organs in Zingiberaceae.

Expression of *AGL6*–like genes in carpel development and ovule formation is considered ancient, but is not conserved during stamen development [93]. Two rice *AGL6*–like genes, *OsMADS6* and *OsMADS17*, are both expressed weakly in stamens [19], [93], [97], while the *AGL6*–like gene *OMADS7* in orchid is expressed in all floral organs except the stamen [80]. The expression of *AhAGL6–like* is confined to floral organs of *A.hainanensis* (Figure 2), which is consistent with *ZinAGL6* in Zingiberales [49] and many other reports in magnoliids [98], [99], Nymphaeles [100], non–grass monocots [101], and Poaceae [93]. Some gymnosperm and eudicot *AGL6* genes are expressed in vegetative tissues, such as *AGL6* from *Arabidopsis*, suggesting expression in the vegetative organs was lost around the origin of the angiosperm clade and regained after the duplication at the base of the core eudicots [102]. It has been considered that the petaloid labellum was derived from two members of the outer androecial whorl in ginger [60]. Deployment of *AhAGL6–like* in the stamen but only faint expression in the labellum implies that *AhAGL6–like* is required to specify stamen development, and low expression of *AhAGL6–like* gene may promote petaloidy in the androecial whorl. This hypothesis is consistent with the results from *Oryza*, in which the flower of *osmads6* mutant presents a decreased number of stamens and the appearance of lodicule–anther mosaic organs [97]. There may be a correlation between the loss of expression of *AGL6* and loss of fertility in the stamen whorl across the Zingiberales [49]. Although suppression of *AGL6–*like genes reduced fertility of stamen and ovules in *Arabidopsis*, the number of stamens did not alter and petaloidy did not occur in the androecial whorl [87], [103].

Both expression patterns of the *AhSEP3* copies in inner whorls are similar with the *SEP3* of *Arabidopsis* and are different from two *ZinSEP3* copies which are expressed across all the floral whorls in Zingiberales. This indicated that *SEP3* genes may act redundantly in these organs regulation and *AhAEP3* genes expression diverse from *ZinSEP3*. In contrast to the *ZinSEP3* expression, *ZinLOFSEP* shows great variability in pattern of selection and expression [49]. Homologous alignment suggests that *AhSEP4* is close to one of the copies *ZinLOFSEP2* in Zingiberales. It is more consistently expressed across all the floral whorls in the species of the ginger families [49]. Unlike *Arabidopsis*, in which the four *SEP* genes are functionally redundant, the rice and orchid *SEP* genes have non–redundant roles during inflorescence development [104], [105]. Compared with rice, the more similar sequences and expression patterns of *SEP* genes in ginger provide additional insights into the hypothesis that *SEP* functions differently in monocots than in eudicots.

During the evolutionary process, the *AP1*, *SEP*, and *AGL6* subfamilies have undergone subfunctionalization and neofunctionalization. These data reveal differential sequences of the four genes may lead to possible functional diversification, their extensive expression in the floral organs indicates that they may act redundantly to control ginger floral architecture and flower development.

### A–E and E1–E2 heterodimers are prevalent in angiosperms

It is well known that many MADS–box proteins form a complex to exert their effect. Protein complexes in various combinations from different species and lineages may provide insight into the functional diversification of these PPIs [44], and formation of different complexes can be the basis of a novel gene regulatory mechanism for the appearance of morphological innovations. Dimerization of MADS–domain proteins is necessary for their capacity to bind their target sequences in DNA [106], and may render them functional in transcriptional activation, further to recruit additional proteins to form a transcriptional complex [86]. We investigated the interactions among AP1/AGL9 proteins from *A.hainanensis* and reconstructed the evolutionary histories of the protein interactions in angiosperms.

The commonly used methods to study protein dimerisation are Y2H, co-immunoprecipitation and surface plasmon resonance *in vitro*, and fluorescence resonance energy transfer (FRET) and BiFC *in vivo*, each has advantages and limitations. BiFC and Y2H were employed to investigate the PPI in *A.hainanensis*, because of low expression level or misfolded/unfolded heterogeneous proteins in the Y2H test system [107], [108], PPI results in yeast are not always conclusive for interactions in *planta*; BiFC enables direct visualization of MADS–box proteins interactions and increases the likelihood that the results reflect the properties of native MADS–box proteins in living cells. The most intriguing aspect of MADS–box PPIs is that interactions between AP1 and SEP proteins are prevalent in monocots and eudicots, *e.g.* AP1–AGL2, AP1–AGL9, AGL2–AGL9, and AGL6–AGL9. The *AP1*/*AGL9* group of MADS–box genes might have evolved through gene duplication from an ancestral gene that contained a paleoAP1 motif [78]; the trace of the possible evolution is retained in the sequences of the *AP1*/*AGL9* group (Figure S1). The tandem arrangement of the *SEP* and *AP1/FUL* genes in genomes also supports a common evolutionary origin of these lineages by an ancient tandem duplication before the origin of extant flowering plants [50], [88]. The conservative interactions may be attributed to the common origin of *SEP* and *AP1*/*FUL*. We tried to determine if the PPIs correlate to the gene duplication and diversifications; however, regardless of paleoAP1 proteins in basal angiosperms or euAP1/euFUL proteins in core eudicots, they are able to interact with the *SEP* lineages. Although gene copy amplification due to duplication was apparently favorable for the plants and manifested in the increase of the interaction complexity [47], the gene diversifications did not block off the capacity to form AP1–SEP (A–E) and SEP–SEP (E1–E2) heterodimers. This indicates that these conservative interactions are necessary for the floral development during the angiosperm evolution; for example, floral initiation and floral organ formation. Two mechanisms of compensation stem from the existence of duplicate genes and from alternative metabolic pathways, regulatory networks, and so on [109]. Thus, the loss of function in one A–E/E1–E2 dimer can be compensated by the other dimer. In contrast, dimmers hardly formed between A and B, B and C, and B and E classes. We reasoned that these proteins do not interact directly, but are capable of participating in multimeric complexes such as ABE or ACE ternary complexes, or even higher–order complexes mediated by A–E and E1–E2 dimers. Additionally, an individual complex comprising A and E (or E and E) proteins has potential for binding to DNA with greater affinity than the A–E or E1–E2 dimer and stabilizing the entire complex.

The ability of AGL9 to form homodimers is likely to have been lost gradually in the core eudicots. In contrast, interactions within the *AP1* lineage and the *AGL2* lineage seem more complex, so we hypothesized that, to a large extent, these interactions evolved multiple times during small–scale and segmental duplications linked to rounds of polyploidization. The difference of precise function for *AP1* lineage members in flowering and floral meristem determination also increases the interaction complexity. Interestingly, the absence of AGL2 homodimers was mostly seen in monocots and basal eudicots, which is contrary to the loss of AGL9 homodimers in core eudicots.

Studies on function of the *AGL6* lineage from *Arabidopsis*, petunia, and rice suggest that the *AGL6*–like genes have a similar function and characteristics as the *SEP* genes, because the two lineage genes are redundant counterparts by duplication [85], [87], [110]. Thus, proteins from the two lineages interact overall with the same partners; AP1 and AGL6 showed a tendency to interact with each other in our interaction and ancestral reconstruction analysis. BiFC and Y2H results showed that AhAGL6–like could form a heterodimer with AhSEP3b, but could not form the homodimer itself, unlike many other *SEP* genes (Figure 3). *AGL6*–like genes could interact with the *AGL9* lineage in all investigated species (Figure 5). The *SEP* lineage has been lost in extant gymnosperms [39], [40]; therefore, we deduced that the PPIs between the *AGL6* and *AGL9* lineage members were established at least no later than divergence between gymnosperms and angiosperms, and likewise for AP1–AGL6. The *AGL6* lineage combination with *FUL* possibly functions in the phase transition of the adult shoot into the reproductive developmental program [102]. Regarding AGL6–AGL6 and AGL2–AGL6, the deduction is drawn by reference to the SEP group, but more evidence is required.

Generally, PPIs for flower development have been quite conservative, with majority pairs were stable and only a few were variable during angiosperm over long evolutionary history of the angiosperms (Figure5I) [18], [46]. Among them, the evolutionarily conservative PPIs have played critical roles in establishing the basic architecture of the flower, while variable PPIs may have contributed to occurrence of floral traits [111].

### *AhFUL* affects the *A.hainanensis* flowering pathway

Transgenic plants ectopically expressing *AP1*–like genes from monocots and eudicots have shown early flowering and (or) *ap1* mutant phenotypes [78], [80], [88], [91], [94], [95], [112]–[114]. Ectopic expression of *AhFUL* in transgenic *Arabidopsis* also led to early flowering and some abnormal floral phenotypes, including the homeotic conversion of the floral organs (Figure 6). We noted that stellate trichomes, which are a feature of rosette and cauline leaves and are seldom observed on sepals of wild–type in *Arabidopsis* [13], appeared on the sepals of the *AhFUL* transgenic plants; the second whorl exhibited short, light green petals. All of these show a floral organ homeotic conversion to sepal–like or leaf–like structures. This further verifies the important role of *AhFUL* in the flowering and the floral organ identity of *A.hainanensis*. However, the *ap1* phenotype, including one or more secondary flowers formed at the base of pistils or at the axils of sepals (Figure 6M, N, O), was not caused by down–regulation of endogenous *AP1* gene. One assumption is that *AhFUL* might form inactive multimeric complexes with the same partners as *AP1* to act as a dominant negative factor, blocking the binding of the functional *AP1* complexes [91]. Flowering induction genes like *FT*, *SOC1*, and *AGL24* are highly expressed in the inflorescence meristem in response to external and internal signals; these proteins in turn promote the expression of the flower meristem identity genes *LFY* and *AP1* [115]. The early flowering phenomena are mainly due to inhibition of the floral repressor *FLC*, and activation of the flowering time genes *FT* and *SOC1*, and the floral initiation genes *LFY* and *AP1* in transgenic *Arabidopsis* plants [101], [116]. Ectopic expression of *AhFUL* led to the up–regulation of *LFY* and *FT* genes associated with the flowering pathway (Figure 7). Therefore, our results suggest that *AhFUL* activated the flowering time genes *FT* in regulating *LFY* activity in transgenic plants, and was attributed to *ap1* phenotype. Although the roles of genes (*CO*/*Hd1*, *FT*/*Hd3a*/*RFT1*, *SOC1*/*OsMADS50*, and *AP1*/*OsMADS14*, *15*, *18*) involved in the photoperiod pathway are generally conserved between rice and *Arabidopsis*, some differences exist. *Hd1* is repressed in long days (LDs) and promoted in short days (SDs) [117]; *Hd3a* acts preferentially in inductive SDs[118]. In addition, rice contains an alternative inductive pathway that activates the *early heading date 1* (*Ehd1*) gene to promote flowering by inducing expression of *Hd3a* and *OsMADS14* [119], [120]. Although differences inevitably exist between evolutionarily distant plant species, we believe that a conserved genetic network controlling photoperiodic flowering is shared in ginger and rice, as well as *Arabidopsis*. Therefore, broad transcription of *AhFUL* may have an ancestral conservative function in promoting flowering, even in regulating sepal and petal identity.

## Supporting Information

Figure S1 **Sequence analysis of** ***AP1/AGL9*** **MADS–box lineage.** (A) Alignment of C–terminal region of AP1/AGL9 lineage MADS–box proteins. We used the C–terminal regions of 59 AP1/AGL9 MADS–box proteins from various plant species and analyzed them with the Clustal W program. The positions of conserved motifs are indicated in the sequences. Identical and similar amino acids are shaded black and grey, respectively. (B) The schematic structure of four *A.hainanensis* AP1/AGL6 proteins.
(TIF)

Figure S2 **Physical protein interaction of AhSEP3b and AhAGL6–like analyses using the BiFC system.** BiFC visualization of the AhSEP3b and AhAGL6–like interaction in transiently co–expressed *Arabidopsis* mesophyll protoplasts. AhSEP3b protein was fused with the C–terminus of YFP and AhAGL6–like protein was fused with the N–terminus of YFP. mCherry–VirD2NLS was included in each transfection to serve as a control for successful transfection as well as for nuclear localization. Empty vectors (pSAT1A–nEYFP–N1/pSAT1A–cEYFP–N1) and expression of AhSEP3b alone (AhSEP3b–pSAT1A–nEYFP–N1/pSAT1A–cEYFP–N1) were used as negative controls.
(TIF)

Figure S3 **Yeast two-hybrid results of AhFUL–AhFUL, AhAGL6–like–AhAGL6–like AhSEP3b–AhAGL6–like and AhSEP3b–AhSEP3b interactions.** A yeast strain (AH109) was transformed with plasmids pGBKT7+pGADT7 (AD×BD, negative control). The ability of the yeast cells transformed with plasmids to grow on synthetic medium lacking tryptophan, leucine, histidine, and adenine, and containing 5 mM 3-amino-1, 2, 4-triazole indicated positive protein–protein interaction.
(TIF)

Figure S4 **Sufficient incubation time is required for sublocalization.** Some combinations localized in cytoplasm after 8 h incubation at 25°C. They localized in the nucleus when the incubation time increased to 16–18 h. Bar  = 20 µm.
(TIF)

Figure S5 **Subcellular localization of GFP as control of** **Figure 4** **.** The fluorescence of protoplasts with pBI221–GFP vector (*35S::GFP*) was observed throughout the cells.
(TIF)

Table S1 **Primers used in this study.**
(DOCX)

Table S2 **Information of genes used in the phylogenetic analysis in** **Figure 1** **.**
(DOCX)

Table S3 **Protein-protein interaction of** ***AP1*** **/** ***AGL9*** **lineage in different species from publication and this study.**
(DOCX)

Table S4 **Matrices for ancestral state reconstructions.** Note: 0 for absence of interaction, 1 for presence of interaction and dash for no experimental record so far.
(DOCX)

Table S5 **Genes cloned in this study.**
(DOCX)

Table S6 **Expression patterns of** ***AP1*** **/** ***AGL9*** **genes comparison in** ***Arabidopsis thaliana*** **,** ***Oryza sativa*** **and** ***Alpinia hainanensis*** **.** Different color represent different plant species, yellow, green and purple represent *Arabidopsis thaliana*, *Oryza sativa* and *Alpinia hainanensis* respectively. E: Genes with known expression in roots, leaves, inflorescence and floral meristems, bract, sepals, petals, stamens, carpels, and fruits are indicated.
(DOCX)
