# Supplementary figures and images for: Functional Conservation and Divergence of Four Ginger AP1/AGL9 MADS–Box Genes Revealed by Analysis of Their Expression and Protein–Protein Interaction, and Ectopic Expression of AhFUL Gene in Arabidopsis
Source: PLoS One. 2014 Dec 2;9(12):e114134. doi: 10.1371/journal.pone.0114134 (PMC4252096; doi:10.1371/journal.pone.0114134)

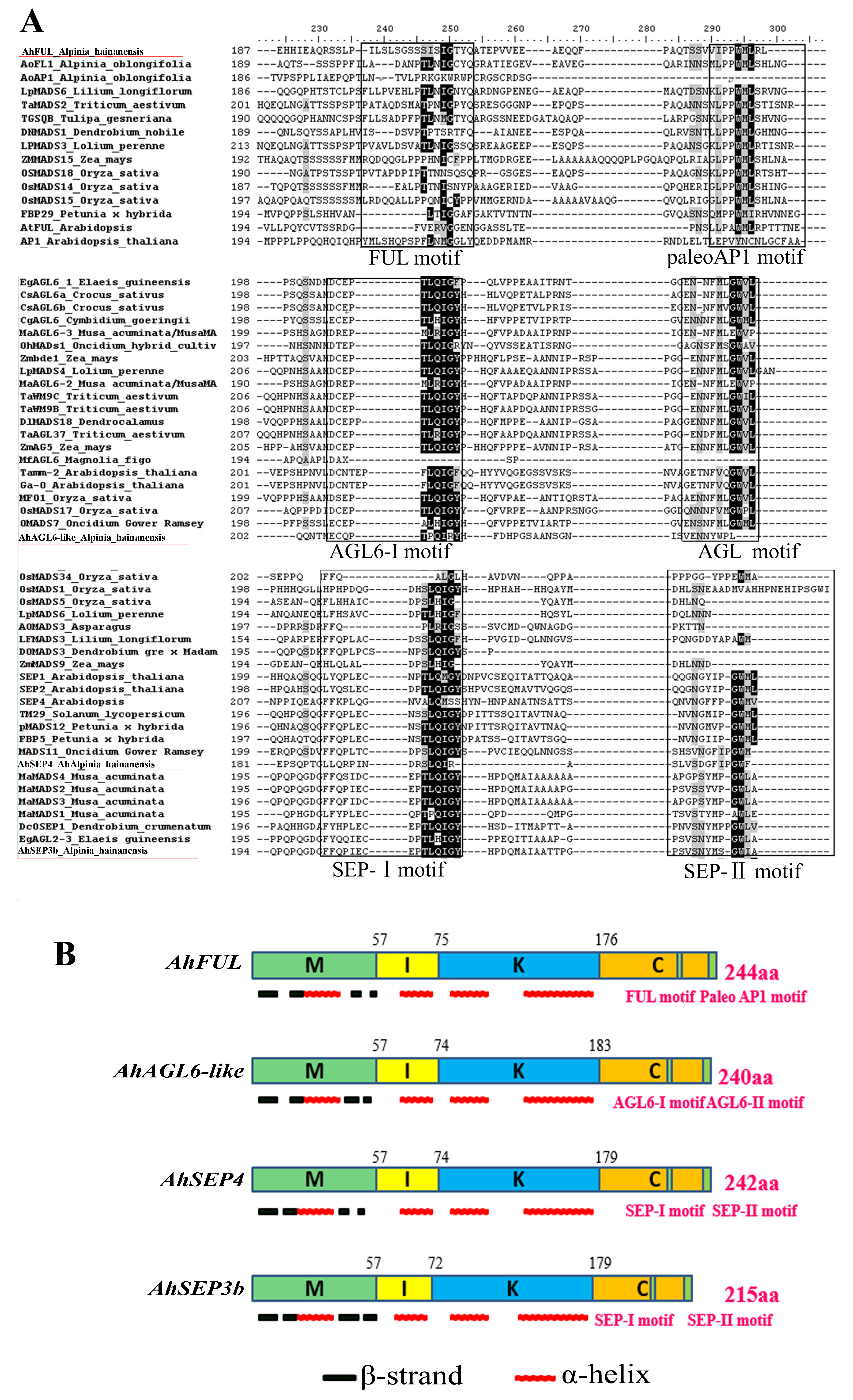

Supplement: Figure S1 — Sequence analysis of AP1/AGL9 MADS–box lineage. (A) Alignment of C–terminal region of AP1/AGL9 lineage MADS–box proteins. We used the C–terminal regions of 59 AP1/AGL9 MADS–box proteins from various plant species and analyzed them with the Clustal W program. The positions of conserved motifs are indicated in the sequences. Identical and similar amino acids are shaded black and grey, respectively. (B) The schematic structure of four A.hainanensis AP1/AGL6 proteins. (TIF) [file pone.0114134.s001.tif]

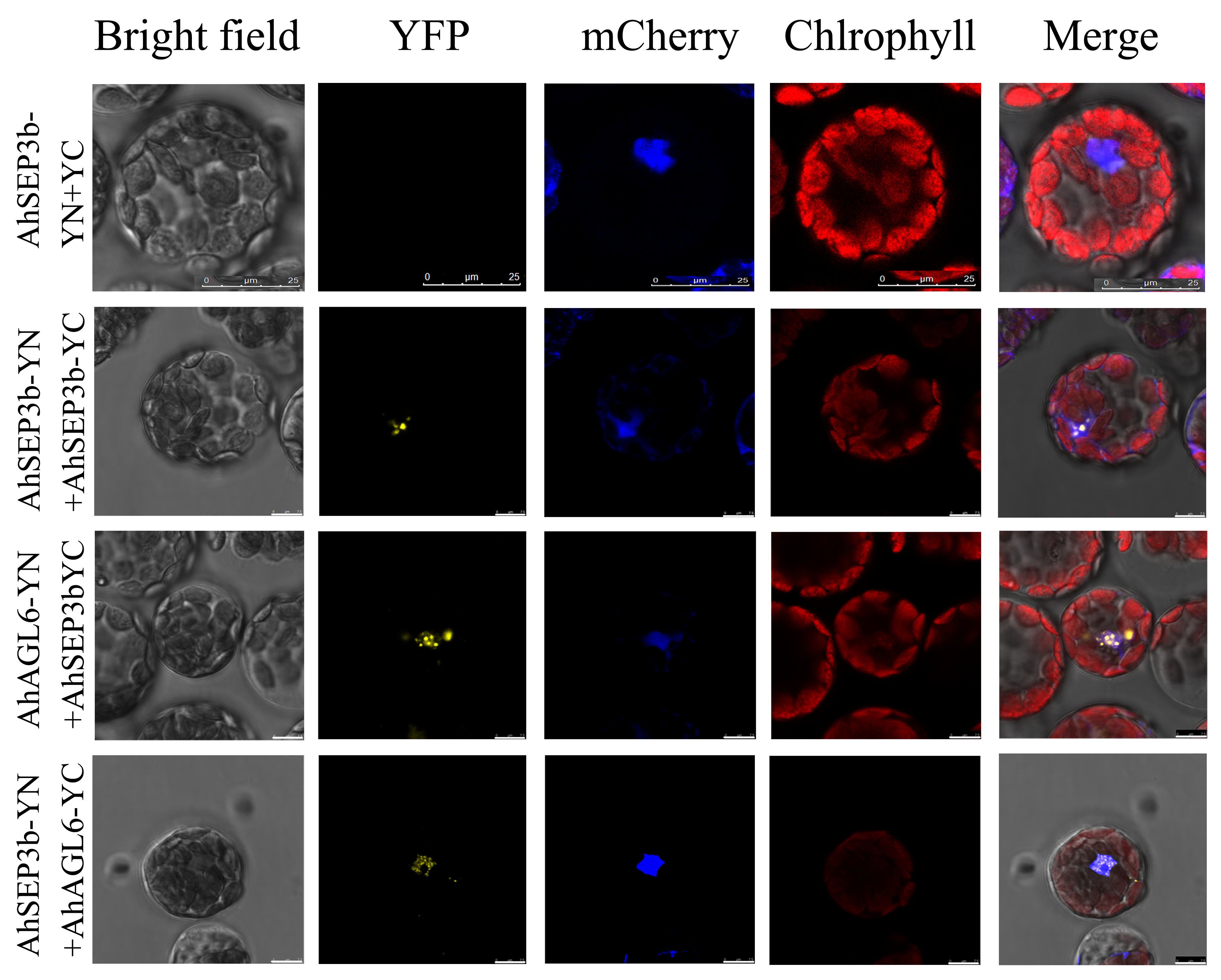

Supplement: Figure S2 — Physical protein interaction of AhSEP3b and AhAGL6–like analyses using the BiFC system. BiFC visualization of the AhSEP3b and AhAGL6–like interaction in transiently co–expressed Arabidopsis mesophyll protoplasts. AhSEP3b protein was fused with the C–terminus of YFP and AhAGL6–like protein was fused with the N–terminus of YFP. mCherry–VirD2NLS was included in each transfection to serve as a control for successful transfection as well as for nuclear localization. Empty vectors (pSAT1A–nEYFP–N1/pSAT1A–cEYFP–N1) and expression of AhSEP3b alone (AhSEP3b–pSAT1A–nEYFP–N1/pSAT1A–cEYFP–N1) were used as negative controls. (TIF) [file pone.0114134.s002.tif]

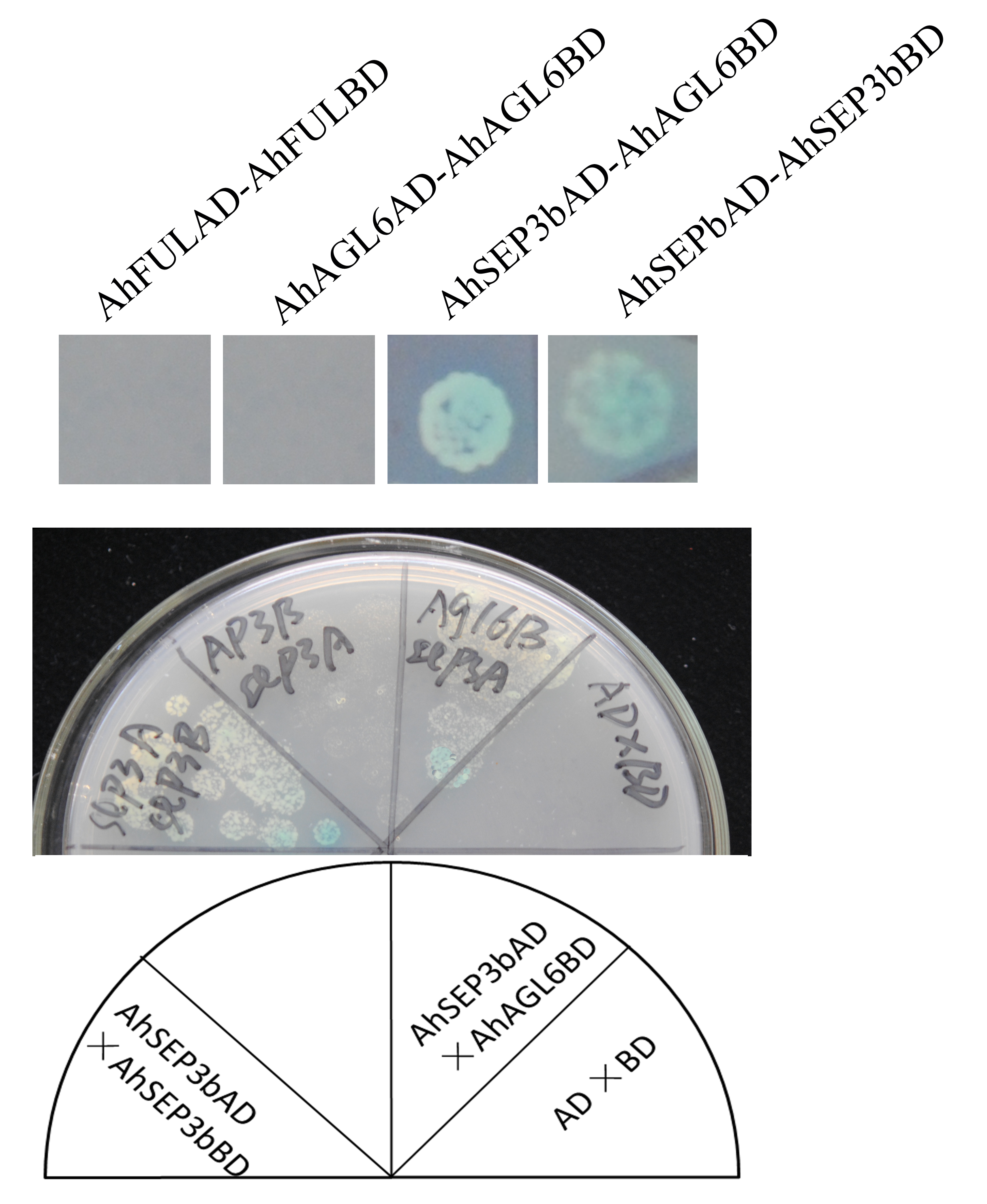

Supplement: Figure S3 — Yeast two-hybrid results of AhFUL–AhFUL, AhAGL6–like–AhAGL6–like AhSEP3b–AhAGL6–like and AhSEP3b–AhSEP3b interactions. A yeast strain (AH109) was transformed with plasmids pGBKT7+pGADT7 (AD×BD, negative control). The ability of the yeast cells transformed with plasmids to grow on synthetic medium lacking tryptophan, leucine, histidine, and adenine, and containing 5 mM 3-amino-1, 2, 4-triazole indicated positive protein–protein interaction. (TIF) [file pone.0114134.s003.tif]

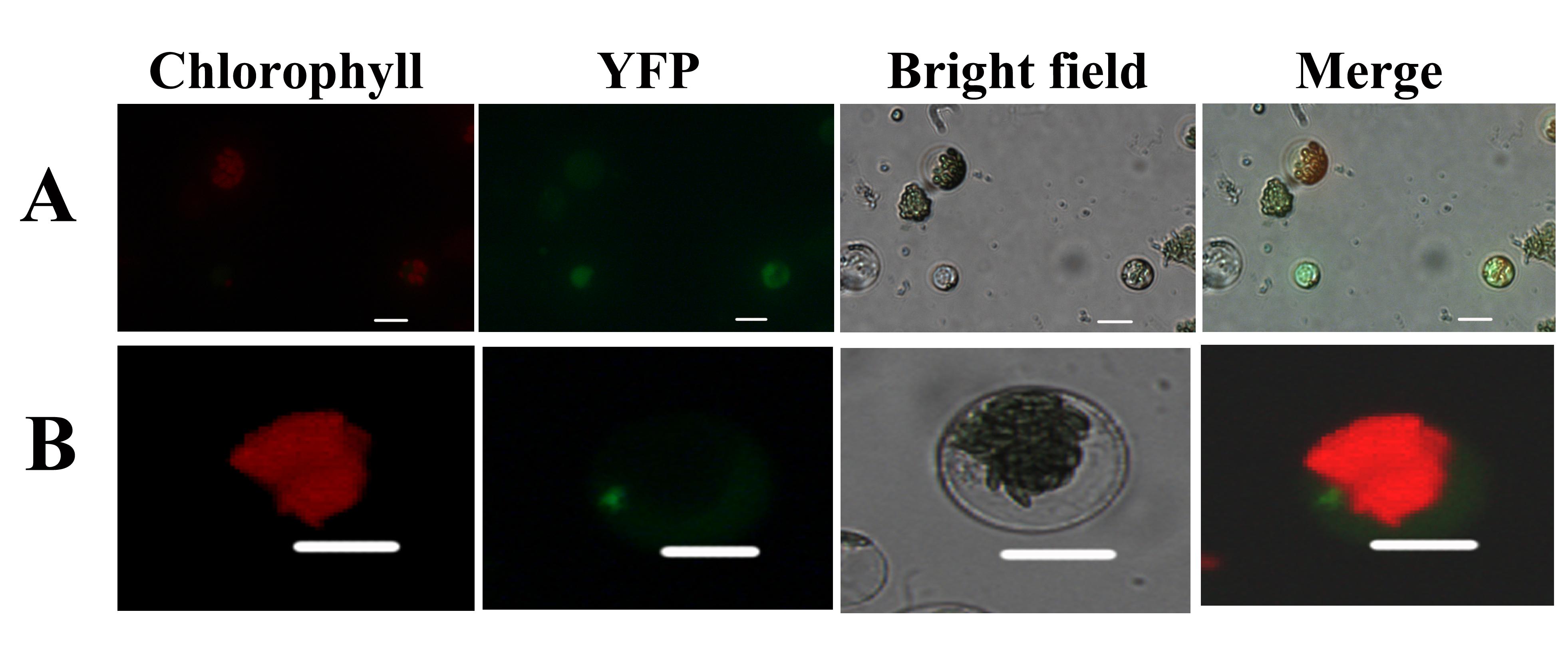

Supplement: Figure S4 — Sufficient incubation time is required for sublocalization. Some combinations localized in cytoplasm after 8 h incubation at 25°C. They localized in the nucleus when the incubation time increased to 16–18 h. Bar = 20 µm. (TIF) [file pone.0114134.s004.tif]

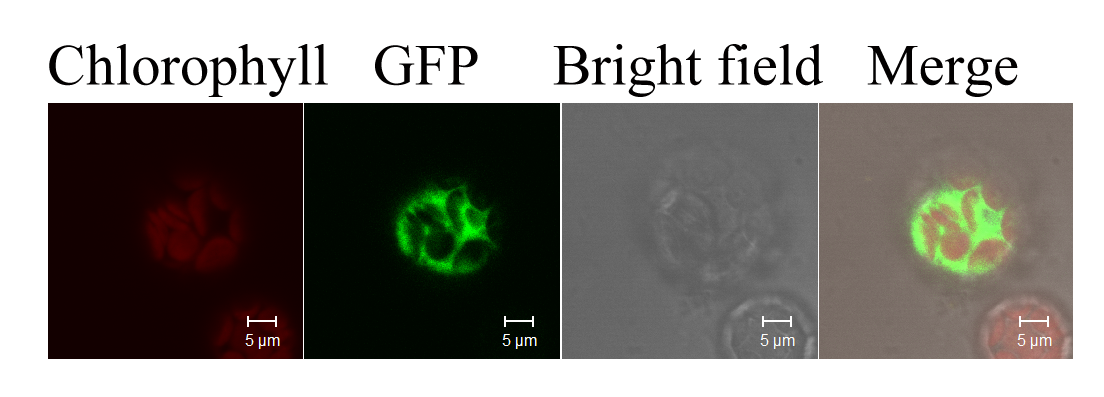

Supplement: Figure S5 — Subcellular localization of GFP as control of Figure 4 . The fluorescence of protoplasts with pBI221–GFP vector (35S::GFP) was observed throughout the cells. (TIF) [file pone.0114134.s005.tif]
