# Supplementary material for: Functional Conservation and Divergence of Four Ginger AP1/AGL9 MADS–Box Genes Revealed by Analysis of Their Expression and Protein–Protein Interaction, and Ectopic Expression of AhFUL Gene in Arabidopsis
Source: PLoS One. 2014 Dec 2;9(12):e114134. doi: 10.1371/journal.pone.0114134 (PMC4252096; doi:10.1371/journal.pone.0114134)
Supplement: Table S1 — Primers used in this study. (DOCX) [file pone.0114134.s006.docx]

**Table S1. Primers used in this study**

| Primer sequences for genes isolation | |
| --- | --- |
| PT | AAGCAGTGGTATCAACGCAGAGTACTTTTTTTTTTTTTTTTTTTTTTTTT |
| RACE-AP | AAGCAGTGGTATCAACGCAGAGTAC |
| MADSSEP-F | ATGGGRAGGGGNVRGGTGGAGSTGAA |
| AhSEP-F | ATGGGGAGGGGTGGGGTGG |
| AhSEP-R | GAGTGAAATTATTTCTTGCTAATAAGAATAG |
| AhSEP-F2 | GATCGAGAACAAGATCAACCG |
| AhSEP-R2 | TTATGCGATCCAACCAGACATGTAG |
| Primer sequences for transgenic plant study | |
| AhFULTR-F | GGTACCATGGGGAGAGGGAGGGT |
| AhFULTR-R | GTCGACTTACAGGCGAAGCATCCAC |
| AhSEP4TR-F | GAGCTCATGGGAAGGGGGAAGGT |
| AhSEP4TR-R | GTCGACTCAAAACCATCCTGGGATG |
| Primer sequences for BiFC assays | |
| AhSEP3bby-F | CG GAATTCG ATGGGGAGGGGTGGGGTG |
| AhSEP3bby-R | TCC CCCGGGTTATGCGATCCAACCAGACATGTAG |
| AhSEP3bbw-F | CCG CTCGAG ATGGGGAGGGGTGGGGTG |
| AhSEP3bbw-R | CG GAATTC TGCGATCCAACCAGACATGTAGTT |
| AhAGL6by-F | ACGC GTCGAC ATGGGGAGAGGGAAGGTGGAG |
| AhAGL6by-R | CG GGATCC TCAAAGAGGCCAATAATTATTCTCG |
| AhAGL6bw-F | ACGC GTCGACATGGGGAGAGGGAAGGTGGAG |
| AhAGL6bw-R | TCC CCCGGG AAGAGGCCAATAATTATTCTCGACC |
| AhSEP4byw-F | ACGC GTCGAC ATGGGAAGGGGGAAGGTGGAG |
| AhSEP4by-R | CG GGATCC TCAAAACCATCCTGGGATGAAGC |
| AhSEP4bw-R | TCC CCCGGG AAACCATCCTGGGATGAAGCCAT |
| AhFULbyw-F | ACGC GTCGAC ATGGGGAGAGGGAGGGTGC |
| AhFULby-R | CG GGATCC TTACAGGCGAAGCATCCACGG |
| AhFULbw-R | TCC CCCGGG CAGGCGAAGCATCCACG |
| Primer sequences for fusing GFP | |
| AhSEP3bGfP-F | CTT GAGCTCATGGGGAGGGGTGGGGTG |
| AhSEP3bGfP-R | GCGC GTCGACTGCGATCCAACCAGACATGTAG |
| AhAGL6GfP-F | CGC GGATCCATGGGGAGAGGGAAGGTGGAG |
| AhAGL6GfP-R | ACGC GTCGACAAGAGGCCAATAATTATTCTCGACC |
| AhSEP4GfP-F | CGC GGATCCATGGGAAGGGGGAAGGT |
| AhSEP4GfP-R | ACGC GTCGACAAACCATCCTGGGATG |
| AhFULGfP-F | CGC GGATCCATGGGGAGAGGGAGGGT |
| AhFULGfP-R | ACGC GTCGAC CAGGCGAAGCATCCAC |
| Primer sequences for relative quantification | |
| AhFULQ-F | GGT CTGCAAGCGCAAAACA |
| AhFULQ-R | CATCCACGGTGGTATAACAACG |
| AhAGL6-F | CGTG TCCAAAGAAAAGAGCGTG |
| AhAGL6-R | CCT GACCGATATGTTCCCAGAAT |
| AhSEP4-F | GACCTAAGCAGATCAGATCCACAA |
| AhSEP4-R | GGACATGAAGCCATCACTCACAA |
| AhSEP3b-F | CTC AGAGAAGGCTGGATGAAAGC |
| AhSEP3b-R | GAGT GCCCTGGTGTTGTTGC |
| Ah18SQ-F | CCAGCATCGGTCGTCTCGTC |
| Ah18SQ-R | GTATGCTTTCGCAGTGGTTCG |
| FTQ-F | TGGGTAAGCAGAGTTGTTGGAGACG |
| FTQ-R | CGCAAGGTTGTTCCAGTTGTAGCAG |
| SOCQ-F | GCACACAAATAGATGAAACGAGGAAAG |
| SOCQ-R | GTGATAAAAACCTAACCAGGAGGAAGC |
| LFYQ-F | CGCTGAAGTGGTTACTGGGACGC |
| LFYQ-R | TCTGGAAGAAGGAACTCACGGCAT |
| AGL24Q-F | AGGTGGTTCTTTGCGATGCTGAT |
| AGL24Q-R | GCACGCTGCTTGGTTTTGTCTT |
| AP1Q-F | CGACCTTACGCCGAAAGACAGC |
| AP1Q-R | CTCCGTTCCCCAAGATAATGCC |
| SEP3Q-F | GGCAGTTAGTAGCCAGCAGGAGTATC |
| SEP3Q-R | GCCGTTTTATTTGTCTCAGTCAGCA |
| UBI-F | CTGTTCACGGAACCCAATTC |
| UBI-R | GGAAAAAGGTCTGACCGACA |
