# Supplementary material for: Functional Conservation and Divergence of Four Ginger AP1/AGL9 MADS–Box Genes Revealed by Analysis of Their Expression and Protein–Protein Interaction, and Ectopic Expression of AhFUL Gene in Arabidopsis
Source: PLoS One. 2014 Dec 2;9(12):e114134. doi: 10.1371/journal.pone.0114134 (PMC4252096; doi:10.1371/journal.pone.0114134)
Supplement: Table S2 — Information of genes used in the phylogenetic analysis in Figure 1 . (DOCX) [file pone.0114134.s007.docx]

**Table S2. Information of 76 genes used in the phylogenetic analysis in Figure 1**

| Order | Family | Species | Accession No. | Name |
| --- | --- | --- | --- | --- |
| Zingiberales | Zingiberaceae | *Alpinia hainanensis* | ? | AhFUL_Alpinia_hainanensis |
| Zingiberales | Zingiberaceae | *Alpinia oblongifolia* | EF521814 | AoFL1_Alpinia_oblongifolia |
| Zingiberales | Zingiberaceae | *Alpinia oblongifolia* | EF521816 | AoAP1_Alpinia_oblongifolia |
| Liliales | Liliaceae | *Lilium longiflorum* | HQ149332 | LpMADS6_Lilium_longiflorum |
| Poales | Poaceae | *Triticum aestivum* | DQ534490 | TaMADS2_Triticum_aestivum |
| Liliales | Liliaceae | *Tulipa gesneriana* | AB472011 | TGSQB_Tulipa_gesneriana |
| Asparagales | Orchidaceae; | *Dendrobium nobile* | EF535599 | DNMADS1_Dendrobium_nobile |
| Poales | Poaceae | *Lolium perenne* | AY198328 | LPMADS3_Lolium_perenne |
| Poales | Poaceae | *Zea mays* | EU963061 | ZMMADS15_Zea_mays |
| Poales | Poaceae | *Oryza sativa* | AF091458 | OSMADS18_Oryza_sativa |
| Poales | Poaceae | *Oryza sativa* | AF058697 | OsMADS14_Oryza_sativa |
| Poales | Poaceae | *Oryza sativa* | AF058698 | OsMADS15_Oryza_sativa |
| Brassicales | Brassicaceae | *Arabidopsis thaliana* | NM_125484 | FUL_Arabidopsis_thaliana |
| Brassicales | Brassicaceae | *Arabidopsis thaliana* | NM_105581 | AP1_Arabidopsis_thaliana |
| Lamiales | Plantaginaceae | *Antirrhinum majus* | X63701 | SQUA_Antirrhinum_majus |
| Solanales | Solanaceae | *Petunia x hybrida* | AF176782 | PFG_Petunia x hybrida |
| Solanales | Solanaceae | *Petunia x hybrida* | AF176783 | FBP26_Petunia x hybrida |
| Brassicales | Brassicaceae | *Arabidopsis thaliana* | NM_113925 | AGL79_Arabidopsis_thaliana |
| Brassicales | Brassicaceae | *Arabidopsis thaliana* | NM_102395 | CAL_Arabidopsis_thaliana |
| Asparagales | Iridaceae | *Crocus sativus* | EF041505 | CsAGL6a_Crocus_sativus |
| Asparagales | Iridaceae | *Crocus sativus* | EF041506 | CsAGL6b_Crocus_sativus |
| Zingiberales | Musaceae | *Musa acuminata* | EU869308 | MaAGL6-3_Musa_acuminata/MusaMADS3 |
| Poales | Poaceae | *Zea mays* | NM_001111862 | Zmbde1_Zea_mays |
| Poales | Poaceae | *Lolium perenne* | AY198329 | LpMADS4_Lolium_perenne |
| Zingiberales | Musaceae | *Musa acuminata* | AY941799 | >MaAGL6-2_Musa acuminata/MusaMADS2 |
| Poales | Poaceae | *Triticum aestivum* | DQ512353 | TaAGL37_Triticum_aestivum |
| Poales | Poaceae | *Zea mays* | NM_001112222 | ZmAG5_Zea_mays |
| Brassicalese | Brassicaceae | *Arabidopsis thaliana* | JX121874 | Tamm-2_Arabidopsis_thaliana |
| Brassicalese | Brassicaceae | *Arabidopsis thaliana* | AFP23748 | Ga-0_Arabidopsis_thaliana |
| Poales | Poaceae | *Oryza sativa* | FJ666318 | MFO1_Oryza_sativa |
| Poales | Poaceae | *Oryza sativa* | FJ668596 | OsMADS17_Oryza_sativa |
| Asparagales | Orchidaceae | *Oncidium Gower Ramsey* | HM140845 | OMADS7_Oncidium_Gower_Ramsey |
| Zingiberales | Musaceae | *Musa acuminata* | GSMUA_Achr2P04350/XP_009384304 | GSMUA_Achr2P04350_Musa_acuminata |
| Zingiberales | Zingiberaceae | *Alpinia hainanensis* | ？ | AhAGL6_Alpinia_hainanensis |
| Poales | Poaceae | *Oryza sativa* | AB003324 | OsMADS34_Oryza_sativa |
| Poales | Poaceae | *Oryza sativa* | L34271/FJ750937 | OsMADS1_Oryza_sativa |
| Poales | Poaceae | *Oryza sativa* | U78890 | OsMADS5_Oryza_sativa |
| Poales | Poaceae | *Lolium perenne* | AY198331 | LpMADS6_Lolium_perenne |
| Asparagales | Asparagaceae | *Asparagus officinalis* | DQ344504 | AOMADS3_Asparagus_officinalis |
| Liliales | Liliaceae | *Lilium longiflorum* | AY826062 | LFMADS3_Lilium_longiflorum |
| Asparagales | Orchidaceae | *Dendrobium gre x Madame Thong-In* | AF198176 | DOMADS3_Dendrobium gre x Madame Thong-In |
| Poales | Poaceae | *Zea mays* | NM_001177865 | ZmMADS9_Zea_mays |
| Brassicales | Brassicaceae | *Arabidopsis thaliana* | NM_121585 | SEP1_Arabidopsis_thaliana |
| Brassicales | Brassicaceae | *Arabidopsis thaliana* | NM_111098 | SEP2_Arabidopsis_thaliana |
| Brassicales | Brassicaceae | *Arabidopsis thaliana* | NM_126418 | SEP4_Arabidopsis_thaliana |
| Solanales | Solanaceae | *Solanum lycopersicum* | AJ302015 | TM29_Solanum_lycopersicum |
| Solanales | Solanaceae | *Petunia x hybrida* | AF335235 | FBP5_Petunia x hybrida |
| Asparagales | Orchidaceae | *Oncidium Gower Ramsey* | HM140847 | MADS11_Oncidium_Gower_Ramsey |
| Solanales | Solanaceae | *Petunia x hybrida* | AF335234 | FBP4_Petunia x hybrida |
| Lamiales | Plantaginaceae | *Antirrhinum majus* | X95467 | DEFH49_Antirrhinum_majus |
| Zingiberales | Musaceae | *Musa acuminata* | XP_009396439 | CAULIFLOWERA_Musa_acuminata |
| Zingiberales | Musaceae | *Musa acuminata* | XP_009411126 | MaMADS5_Musa_acuminata |
| Zingiberales | Zingiberaceae | *Elettariopsis smithiae* | AHC54088 | LOFSEP_Elettariopsis_smithiae |
| Liliales | Alstroemeriaceae | *Alstroemeria ligtu* | BAM34478 | AlMADS_Alstroemeria_ligtu |
| Asparagales | Orchidaceae | *Phalaenopsis equestris* | AHW52536 | SEP1_Phalaenopsis_equestris |
| Solanales | Solanaceae | *Solanum lycopersicum* | AY294329 | LeMADS1_Solanum_ lycopersicum |
| Solanales | Solanaceae | *Petunia x hybrida* | AF335236 | FBP9_Petunia |
| Solanales | Solanaceae | *Petunia x hybrida* | AF335241 | FBP23_Petunia |
| Zingiberales | Zingiberaceae | *Alpinia hainanensis* | ？ | AhSEP4_Alpinia_hainanensis |
| Zingiberales | Musaceae | *Musa acuminata* | EU869309 | MaMADS4_Musa_acuminata |
| Zingiberales | Musaceae | *Musa acuminata* | EU869306 | MaMADS2_Musa_acuminata |
| Zingiberales | Musaceae | *Musa acuminata* | AY941800 | MaMADS3_Musa_acuminata |
| Zingiberales | Musaceae | *Musa acuminata* | EU869307 | MaMADS1_Musa_acuminata |
| Asparagales | Orchidaceae | *Dendrobium crumenatum* | DQ119842 | DcOSEP1_Dendrobium_crumenatum |
| Brassicales | Brassicaceae | *Arabidopsis thaliana* | NM_102272 | SEP3_Arabidopsis_thaliana |
| Poales | Poaceae | *Oryza sativa* | U78891 | OsMADS7_Oryza_sativa |
| Poales | Poaceae | *Oryza sativa* | U78892 | OsMADS8_Oryza_sativa |
| Solanales | Solanaceae | *Petunia x hybrida* | M91666 | FBP2_Petunia x hybrida |
| Lamiales | Plantaginaceae | *Antirrhinum majus* | X95469 | DEFH200_Antirrhinum_majus |
| Lamiales | Plantaginaceae | *Antirrhinum majus* | X95468 | DEFH72_Antirrhinum_majus |
| Zingiberales | Zingiberaceae | *Alpinia hainanensis* | ？ | AhSEP3b_Alpinia_hainanensis |
| Zingiberales | Zingiberaceae | *Alpinia hainanensis* | FJ861327 | AhSEP3_Alpinia_hainanensis |
| Brassicales | Brassicaceae | *Arabidopsis thaliana* | NM_001203767 | STK_Arabidopsis_thaliana |
| Brassicales | Brassicaceae | *Arabidopsis thaliana* | AY727623 | SHP1_Arabidopsis_thaliana |
| Brassicales | Brassicaceae | *Arabidopsis thaliana* | AY727647 | SHP2_Arabidopsis_thaliana |
| Poales | Poaceae | *Oryza sativa* | AF151693 | OsMADS13_Oryza_sativa |
