# Supplementary material for: Functional Conservation and Divergence of Four Ginger AP1/AGL9 MADS–Box Genes Revealed by Analysis of Their Expression and Protein–Protein Interaction, and Ectopic Expression of AhFUL Gene in Arabidopsis
Source: PLoS One. 2014 Dec 2;9(12):e114134. doi: 10.1371/journal.pone.0114134 (PMC4252096; doi:10.1371/journal.pone.0114134)
Supplement: Table S3 — Protein-protein interaction of AP1 / AGL9 lineage in different species from publication and this study. (DOCX) [file pone.0114134.s008.docx]

**Table S3. Protein-protein interaction of *AP1*/*AGL9* lineage in different species from publication and this study**

| Order | Family | Species | Protein | Lineage | Interacting partners | References |  |
| --- | --- | --- | --- | --- | --- | --- | --- |
| asparagales | Iridaceae | *Crocus sativus.* | paleoAP1 | paleoAP1 | CsSEP3 | (Tsaftaris, Pasentsis et al. 2011) |  |
|  |  |  | paleoAP1 | paleoAP1 | CsAP3 | (Tsaftaris, Pasentsis et al. 2011) |  |
| poales | Poaceae | *Oryza sativa* | OsMADS7 | SEP3 | OsMADS7 OsMADS8 OsMADS1 | (Cui, Han et al. 2010) |  |
|  |  |  | OsMADS8 | SEP3 | OsMADS7 OsMADS8 OsMADS1 | (Cui, Han et al. 2010) |  |
|  |  |  | OsMADS5 | SEP1/2/4 |  | (Cui, Han et al. 2010) |  |
|  |  |  | OsMADS1 | SEP1/2/4 | OsMADS1 OsMADS7 OsMADS8 | (Cui, Han et al. 2010) |  |
|  |  |  | OsMADS14 | paleoAP1 | OsMADS13 OsMADS1 OsMADS7 | (Lim et al. 2000; Favaro et al. 2002; Cooper et al. 2003) |  |
|  |  |  | OsMADS15 | paleoAP1 | OsMADS1 OsMADS7 | (Lim et al. 2000; Cooper et al. 2003) |  |
|  |  |  | OsMADS18 | paleoAP1 | OsMADS7 OsMADS8 OsMADS6 OsMADS47 | (Cooper et al. 2003; Fornara et al. 2004) |  |
|  |  |  | OsMADS1 | SEP1/2/4 | OsMADS14 OsMADS15 OsMADS7 | (Lim et al. 2000; Cooper et al. 2003) |  |
|  |  |  | OsMADS5 | SEP1/2/4 | OsMADS13 | (Cooper et al. 2003) |  |
|  |  |  | OsMADS7 | SEP3 | OsMADS14 OsMADS15 OsMADS18 OsMADS3 OsMADS13 OsMADS1 | (Favaro et al. 2002; Cooper et al. 2003; Fornara et al. 2004) |  |
|  |  |  | OsMADS8 | SEP3 | OsMADS18 OsMADS3 OsMADS13 OsMADS16 | (Favaro et al. 2002; Cooper et al. 2003; Lee et al. 2003; Fornara et al. 2004) |  |
|  |  |  | OsMADS6 | AGL6 | OsMADS1 OsMADS7, OsMADS8 | Moon et al., 1999 Seok et al., 2010) |  |
| poales | Poaceae | *Phyllostachys praecox* | PpMADS1 | paleoAP1 | AP1 | (Lin, Peng et al. 2009) |  |
|  |  |  | PpMADS2 | paleoAP1 | AP1 | (Lin, Peng et al. 2009) |  |
| poales | Poaceae | *Lolium perenne* | LpMADS1 | paleoAP1 | LpMADS1 LpMADS2 | (Ciannamea et al. 2006) |  |
|  |  |  | LpMADS2 | paleoAP1 | LpMADS1 LpMADS2 LpMADS3 | (Ciannamea et al. 2006) |  |
|  |  |  | LpMADS3 | paleoAP1 | LpMADS2 | (Ciannamea et al. 2006) |  |
| poales | Poaceae | *Triticum aestivum* | WAP1 | paleoAP1 | WSEP-A WSEP-B WSEP-D | (Shitsukawa et al. 2007) |  |
|  |  |  | WLHS1-B | SEP1/2/4 | WLHS1-B WLHS1-D WSEP-A WSEP-B WSEP-D | (Shitsukawa et al. 2007) |  |
|  |  |  | WLHS1-D | SEP1/2/4 | WLHS1-B WLHS2-D WSEP-A WSEP-B WSEP-D | (Shitsukawa et al. 2007) |  |
|  |  |  | WSEP-A | SEP3 | WAP1 WAG-1 WAG-2 WLHS1-B WLHS1-D WSEP-A WSEP-B WSEP-D | (Shitsukawa et al. 2007) |  |
|  |  |  | WSEP-B | SEP3 | WAP1 WAG-1 WAG-2 WLHS1-B WLHS2-D WSEP-A WSEP-B WSEP-D | (Shitsukawa et al. 2007) |  |
|  |  |  | WSEP-D | SEP3 | WAP1 WAG-1 WAG-2 WLHS1-B WLHS3-D WSEP-A WSEP-B WSEP-D | (Shitsukawa et al. 2007) |  |
| Asteridae | Asteraceae | *Dendrathema grandiflorum* | CDM111 | euAP1 | CDM44 | (Shchennikova et al. 2004) |  |
|  |  |  | CDM41 | AGL79 | CDM44 | (Shchennikova et al. 2004) |  |
|  |  |  | CDM8 | euFUL | CDM44 | (Shchennikova et al. 2004) |  |
|  |  |  | CDM44 | SEP3 | CDM111 CDM8 CDM41 CDM37 CDM19 CDM86 | (Shchennikova etal. 2004) |  |
| Asterales | Asteraceae | *Gerbera hybrida* | GRCD1 | SEP3 | GAGA1 GAGA2 | (Kotilainen et al. 2000) |  |
|  |  |  | GRCD2 | SEP1/2 | GGLO1 GAGA1 GAGA2 | (Uimari et al. 2004) |  |
|  |  |  | GGLO1 | PI | GRCD2 GDEF1 GDEF2 | (Uimari et al. 2004;Ruokolainen, Ng et al. 2010) |  |
|  |  |  | GRCD3 | AGL6 | GGLO1 GAGA1 GAGA2 GRCD1 GRCD2 GRCD3 GRCD4 GRCD5 GSQUA2 GSQUA3 GSQUA5 | (Ruokolainen, Ng et al. 2010) |  |
|  |  |  | GRCD4 | SEP1/2/4 | GGLO1 GAGA1 GAGA2 GRCD1 GRCD2 GRCD3 GRCD4 GRCD5 GSQUA1 GSQUA2 GSQUA3 GSQUA5 | (Ruokolainen, Ng et al. 2010) |  |
|  |  |  | GRCD5 | SEP3 | GGLO1 GAGA1 GAGA2 GRCD1 GRCD2 GRCD3 GRCD4 GRCD5 GSQUA1 GSQUA2 GSQUA3 GSQUA5 | (Ruokolainen, Ng et al. 2010) |  |
|  |  |  | GSQUA1 | euAP1 | GGLO1 GAGA1 GAGA2 GRCD1 GRCD2 GRCD3 GRCD4 GRCD5 GSQUA2 GSQUA3 GSQUA5 | (Ruokolainen, Ng et al. 2010) |  |
|  |  |  | GSQUA2 | paleoAP1 | GGLO1 GAGA1 GAGA2 GRCD1 GRCD2 GRCD3 GRCD4 GRCD5 GSQUA2 GSQUA3 GSQUA5 | (Ruokolainen, Ng et al. 2010) |  |
| Solanum | Lycopersicum | *Solanum lycopersicum* | MC | euAP1 | SLMBP21 RIN | (Leseberg et al. 2008) |  |
|  |  |  | TM4 | euFUL | RIN | (Leseberg et al. 2008) |  |
|  |  |  | SLMBP7 | euFUL | SLMBP21 LeMADS1 RIN TAG1 TM5 | (Leseberg et al. 2008) |  |
|  |  |  | SLMBP20 | AGL79 | RIN | (Leseberg et al. 2008) |  |
|  |  |  | TM5 | SEP3 | SLMBP7 TAG1 TSTK TAGL1 SLMBP3 | (de Martino et al. 2006; Leseberg et |  |
|  |  |  | TM29 | SEP1/2 | TAG1 TAGL1 SLMBP21 RIN MC TM4 SLMBP20 SLMBP7 LePI | (Busi et al. 2003; Leseberg et al. 2008) |  |
|  |  |  | RIN | SEP4 | TAG1 TSTK SLMBP3 TM29 SLMBP21 LeMADS1 | (Leseberg et al. 2008) |  |
| Solanum | Llycopersicum | *Petunia hybrida* | phAGL6 | AGL6 | PMADS3 FBP6 FBP7 FBP11 FBP2 FBP5 pMADS12 FBP4 FBP9 FBP23 PhAGL6 UNS FBP21 FBP22 FBP28 | (Rijpkema, Zethof et al. 2009) |  |
|  |  |  | FBP2 | SEP3 | PMASD3 FBP6 FBP7 FBP11 FBP2 FBP5 pMADS12 FBP4 FBP9 FBP23 PhAGL6 UNS FBP21 FBP22 FBP28 | (Rijpkema, Zethof et al. 2009) | |
|  |  |  | FBP2 | SEP3 | FBP26 FBP29 pMADS3 FBP6 FBP7 FBP11 FBP23 | (Favaro et al. 2002; Immink and Angenent 2002; Kapoor et al. 2002;Ferrario et al. 2003; Immink et al. 2003) |  |
|  |  |  | FBP26 | euFUL | FBP29 FBP6 FBP23 FBP2 | (Immink et al. 2003) |  |
|  |  |  | PFG | euFUL | FBP29 FBP4 FBP5 FBP23 | (Immink et al. 2003) |  |
|  |  |  | FBP29 | AGL79 | FBP26 PFG FBP5 FBP23 FBP2 | (Ferrario et al. 2003; Immink et al. 2003) |  |
|  |  |  | FBP5 | SEP1/2 | PFG FBP29 pMADS3 FBP6 FBP7 FBP11 FBP23 | (Favaro et al. 2002; Immink and Angenent 2002; Ferrario et al. 2003;Immink et al. 2003) |  |
|  |  |  | pMADS12 | SEP1/2 | FBP6 FBP11 | (Ferrario et al. 2003) |  |
|  |  |  | FBP4 | SEP4 | PFG FBP4 | (Immink et al. 2003) |  |
| Fabales | Fabaceae | Glycine_max | GmMADS28 | SEP3 | GmSIP1 GmSIP2 GmSIP3 GmSIP4 | (Huang et al., 2014) |  |
| Lamiales | Plantaginaceae | *Antirrhinum majus* | SQUA | euAP1 | SQUA FAR PLE DEFH200 DEFH72 | (Davies et al. 1996; Davies et al. 1999; Egea-Cortines, Saedler, and Sommer 1999; Causier, Cook, and Davies 2003) |  |
|  |  |  | DEFH49 | SEP1/2 | FAR PLE | (Davies et al. 1996; Davies et al. 1999) |  |
|  |  |  | DEFH200 | SEP3 | SQUA FAR PLE | (Davies et al. 1996; Davies et al. 1999; Causier, Cook, and Davies 2003) |  |
|  |  |  | DEFH72 | SEP3 | SQUA PLE | (Davies et al. 1996; Davies et al. 1999; Causier, Cook, and Davies 2003) |  |
| Brassicalese | Brassicaceae | *Arabidopsis thaliana* | AGL6 | AGL6 | SEP | de Folter, S., Immink, R.G.H., Kieffer, M. et al. (2005) |  |
|  |  |  | FUL | euFUL | AG SEP1 SEP3 SEP4 | (de Folter et al. 2005) |  |
|  |  |  | SEP1 | SEP1/2 | AP1 FUL AG SHP1 SHP2 STK SEP4 | (Fan et al. 1997; Fornara et al. 2004; de Folter et al. 2005; He et al. 2007) |  |
|  |  |  | SEP2 | SEP1/2 | AG SEP4 | (Fan et al. 1997; de Folter et al. 2005; He et al. 2007) |  |
|  |  |  | SEP4 | SEP4 | AP1 FUL SEP4 | (de Folter et al. 2005; He et al. 2007) |  |
|  |  |  | AP1 | euAP1 | SEP1 SEP3 SEP4 | (Honma and Goto 2001; Pelaz et al. 2001; Fornara et al. 2004; de Folter et al. 2005) |  |
|  |  |  | SEP3 | SEP3 | AP1 CAL FUL AG SHP1 SHP2 STK SEP4 | (Fan et al. 1997; Honma and Goto 2001; Pelaz et al. 2001; Favaro et al2003; Fornara et al. 2004; de Folter et al. 2005; He et al. 2007). |  |
| Rosidae | Brassicaceae | *Brassica oleracea var.botrytis* | BoCAL | euAP1 | BoCAL BoAP1 | (An et al. 2007) |  |
|  |  |  | BoAP1 | euAP1 | BoCAL | (An et al. 2007) |  |
| Buxales | Buxaceae | *Pachysandra terminalis* | PAteFL1 | FUL | PAteFL1 PAteFL3 PAteAG PAteSEP3_1 PAteSEP3_2 | (Liu, Zhang et al. 2010) |  |
|  |  |  | PAteFL3 | FUL | PAteFL1 PAteFL3 PAteAG PAteSEP3_1 | (Liu, Zhang et al. 2010) |  |
|  |  |  | PAteSEP3_1 | SEP3 | PAteFL1 PAteFL3 PAteAG PAteSEP3_2 | (Liu, Zhang et al. 2010) |  |
|  |  |  | PAteSEP3_2 | SEP3 | PAteFL1 PAteFL3 PAteAG PAteSEP3_1 PAteSEP3_2 | (Liu, Zhang et al. 2010) |  |
|  |  |  |  |  |  | (Liu, Zhang et al. 2010) |  |
| Ranunculales | Lardizabalaceae | *Akebia trifoliata* | AktFL1 | FUL | AktFL1 AktFL2 AktAG1 AktAG2 AktPI AktSEP3 AktSEP1_1 AktSEP1_2 | (Liu, Zhang et al. 2010) |  |
|  |  |  | AktFL2 | FUL | AktFL1 AktFL2 AktAG1 AktAG2 AktSEP3 AktSEP1_1 AktSEP1_2 | (Liu, Zhang et al. 2010) |  |
|  |  |  | AktSEP1_1 | SEP1/2/4 | AktFL1 AktFL2 AktAG1 AktAG2 AktSEP3 AktSEP1_1 AktSEP1_2 | (Liu, Zhang et al. 2010) |  |
|  |  |  | AktSEP1_2 | SEP1/2/4 | AktFL1 AktFL2 AktAG1 AktAG2 AktSEP3 AktSEP1_1 AktSEP1_2 | (Liu, Zhang et al. 2010) |  |
|  |  |  | AktSEP3 | SEP3 | AktFL1 AktFL2 AktAG1 AktAG2 AktSEP3 AktSEP1_1 AktSEP1_2 | (Liu, Zhang et al. 2010) |  |
| Ranunculales | Eupteleaceae | *Euptelea pleiospermum* | EUplFL1 | FUL | EUplSEP1 EUplSEP3 | (Liu, Zhang et al. 2010) |  |
|  |  |  | EUplFL2 | FUL | EUplSEP1 EUplSEP3 | (Liu, Zhang et al. 2010) |  |
|  |  |  | EUplSEP1 | SEP1/2/4 | EUplFL1 EUplFL2 EUplAG2 EUplSEP3 | (Liu, Zhang et al. 2010) |  |
|  |  |  | EUplSEP3 | SEP3 | EUplFL1 EUplFL2 EUplAG1 EUplAG2 EUplSEP1 EUplSEP3 | (Liu, Zhang et al. 2010) |  |
| Chloranthales | Chloranthaceae | *Chloranthus spicatus* | CsAP3 | paleoAP3 | CsPI | (Su et al. 2008) |  |
|  |  |  | CsPI | PI | CsAP3 | (Su et al. 2008) |  |
|  |  |  | CsAP1 | paleoAP1 | CsAP1 CsSEP3 CsSEP1/2 | (Liu, Zhang et al. 2010) |  |
| Ranunculales | Ranunculaceae | *Aquilegia coerulea* | AqcFL1 | paleoAP1 | AqcAGL6 AqcSOC AqcSEP1 AqcSEP2A AqcSEP3 AqcAGL24 | (Pabon-Mora, Sharma et al. 2013) |  |
| Ranunculales | Berberidaceae | *Epimedium_sagittatum* | EsFUL | paleoAP1 | EsAGL6 EsAG EsAP3 EsAGL2 EsAG11 | (Sun, Huang et al. 2014) |  |
| Asparagales | Orchidaceae | *Phalaenopsis_equestris* | PeSEP1 | SEP3 | PeMADS1 PeMADS2 PeMADS3 PeMADS4 PeMADS5 PeMADS6 PeMADS7 | (Pan, Chen et al. 2014) |  |
|  |  |  | PeSEP2 | SEP1/2/4 | PeMADS1 PeMADS2 PeMADS3 PeMADS4 PeMADS5 PeMADS6 PeMADS7 | (Pan, Chen et al. 2014) |  |
|  |  |  | PeSEP3 | SEP3 | PeMADS1 PeMADS2 PeMADS3 PeMADS4 PeMADS5 PeMADS6 PeMADS7 | (Pan, Chen et al. 2014) |  |
|  |  |  | PeSEP4 | SEP4 | PeMADS1 PeMADS2 PeMADS3 PeMADS4 PeMADS5 PeMADS6 PeMADS7 | (Pan, Chen et al. 2014) |  |
| Zingiberales | Zingiberaceae | *Alpinia hainanensis* | AhFUL | paleoAP1 | AhSEP3b AhSEP4 AhAGL6 AhFUL AhAG AhAP3 AhPI | This study |  |
|  |  |  | AhSEP3b | SEP3 | AhSEP3b AhSEP4 AhAGL6 AhFUL AhAG AhAP3 AhPI | This study |  |
|  |  |  | AhSEP4 | SEP1/2 | AhSEP3b AhSEP4 AhAGL6 AhFUL AhAG AhAP3 AhPI | This study |  |
|  |  |  | AhAGL6 | AGL6 | AhSEP3b AhSEP4 AhAGL6 AhFUL AhAG AhAP3 AhPI | This study |  |

**Reference**

An, Y. H., X. F. Li, J. X. Zhu, X. H. Shao, Y. Sun and L. J. Xiong (2007). "The screening of interaction factors with BoCAL and BoAP1 related to curd formation." Fen zi xi bao sheng wu xue bao = Journal of molecular cell biology / Zhongguo xi bao sheng wu xue xue hui zhu ban **40**(2): 130-136.

Busi, M. V., C. Bustamante, C. D'Angelo, M. Hidalgo-Cuevas, S. B. Boggio, E. M. Valle and E. Zabaleta (2003). "MADS-box genes expressed during tomato seed and fruit development." Plant Molecular Biology **52**(4): 801-815.

Causier, B., H. Cook and B. Davies (2003). "An Antirrhinum ternary complex factor specifically interacts with C-function and SEPALLATA-like MADS-box factors." Plant Molecular Biology **52**(5): 1051-1062.

Ciannamea, S., K. Kaufmann, M. Frau, I. A. Tonaco, K. Petersen, K. K. Nielsen, G. C. Angenent and R. G. Immink (2006). "Protein interactions of MADS box transcription factors involved in flowering in Lolium perenne." J Exp Bot **57**(13): 3419-3431.

Cooper, B. (2003). "A network of rice genes associated with stress response and seed development." Proceedings of the National Academy of Sciences **100**(8): 4945-4950.

Cui, R., J. Han, S. Zhao, K. Su, F. Wu, X. Du, Q. Xu, K. Chong, G. Theissen and Z. Meng (2010). "Functional conservation and diversification of class E floral homeotic genes in rice (Oryza sativa)." Plant Journal **61**(5): 767-781.

Davies, B., M. EgeaCortines, E. D. Silva, H. Saedler and H. Sommer (1996). "Multiple interactions amongst floral homeotic MADS box proteins." Embo Journal **15**(16): 4330-4343.

Davies, B., P. Motte, E. Keck, H. Saedler, H. Sommer and Z. Schwarz-Sommer (1999). "PLENA and FARINELLI: redundancy and regulatory interactions between two Antirrhinum MADS-box factors controlling flower development." Embo Journal **18**(14): 4023-4034.

de Folter, S., R. G. H. Immink, M. Kieffer, L. Parenicova, S. R. Henz, D. Weigel, M. Busscher, M. Kooiker, L. Colombo, M. M. Kater, B. Davies and G. C. Angenent (2005). "Comprehensive interaction map of the Arabidopsis MADS box transcription factors." Plant Cell **17**(5): 1424-1433.

de Martino, G., I. Pan, E. Emmanuel, A. Levy and V. F. Irish (2006). "Functional analyses of two tomato APETALA3 genes demonstrate diversification in their roles in regulating floral development." Plant Cell **18**(8): 1833-1845.

Egea-Cortines, M., H. Saedler and H. Sommer (1999). "Ternary complex formation between the MADS-box proteins SQUAMOSA, DEFICIENS and GLOBOSA is involved in the control of floral architecture in Antirrhinum majus." Embo Journal **18**(19): 5370-5379.

Fan, H. Y., Y. Hu, M. Tudor and H. Ma (1997). "Specific interactions between the K domains of AG and AGLs, members of the MADS domain family of DNA binding proteins." Plant Journal **12**(5): 999-1010.

Favaro, R., R. Immink, V. Ferioli, B. Bernasconi, M. Byzova, G. Angenent, M. Kater and L. Colombo (2002). "Ovule-specific MADS-box proteins have conserved protein-protein interactions in monocot and dicot plants." Molecular Genetics and Genomics **268**(2): 152-159.

Ferrario, S. (2003). "The MADS Box Gene FBP2 Is Required for SEPALLATA Function in Petunia." The Plant Cell Online **15**(4): 914-925.

Fornara, F. (2004). "Functional Characterization of OsMADS18, a Member of the AP1/SQUA Subfamily of MADS Box Genes." Plant Physiology **135**(4): 2207-2219.

He, C., H. Sommer, B. Grosardt, P. Huijser and H. Saedler (2007). "PFMAGO, a MAGO NASHI-like factor, interacts with the MADS-domain protein MPF2 from Physalis floridana." Molecular Biology and Evolution **24**(5): 1229-1241.

Honma, T. and K. Goto (2001). "Complexes of MADS-box proteins are sufficient to convert leaves into floral organs." Nature **409**(6819): 525-529.

Huang, F., G. Xu, Y. Chi, H. Liu, Q. Xue, T. Zhao, J. Gai and D. Yu (2014). "A soybean MADS-box protein modulates floral organ numbers, petal identity and sterility." Bmc Plant Biology **14**.

Immink, R. G. H., S. Ferrario, J. Busscher-Lange, M. Kooiker, M. Busscher and G. C. Angenent (2003). "Analysis of the petunia MADS-box transcription factor family." Molecular Genetics and Genomics **268**(5): 598-606.

Jeongsim Lim, Y.-H. M., Gynheung An and Sung Key Jang (2000). "Two rice MADS domain proteins interact with OsMADS1." Plant Molecular Biology **44**(4): 513-527.

Kapoor, M., S. Tsuda, Y. Tanaka, T. Mayama, Y. Okuyama, S. Tsuchimoto and H. Takatsuji (2002). "Role of petunia pMADS3 in determination of floral organ and meristem identity, as revealed by its loss of function." Plant Journal **32**(1): 115-127.

Kotilainen, M. (2000). "GRCD1, an AGL2-like MADS Box Gene, Participates in the C Function during Stamen Development in Gerbera hybrida." The Plant Cell Online **12**(10): 1893-1902.

Lee, S., J. S. Jeon, K. An, Y. H. Moon, S. Lee, Y. Y. Chung and G. An (2003). "Alteration of floral organ identity in rice through ectopic expression of OsMADS16." Planta **217**(6): 904-911.

Leseberg, C. H., C. L. Eissler, X. Wang, M. A. Johns, M. R. Duvall and L. Mao (2008). "Interaction study of MADS-domain proteins in tomato." Journal of Experimental Botany **59**(8): 2253-2265.

Lin, E.-P., H.-Z. Peng, Q.-Y. Jin, M.-J. Deng, T. Li, X.-C. Xiao, X.-Q. Hua, K.-H. Wang, H.-W. Bian, N. Han and M.-Y. Zhu (2009). "Identification and characterization of two Bamboo (Phyllostachys praecox) AP1/SQUA-like MADS-box genes during floral transition." Planta **231**(1): 109-120.

Liu, C., J. Zhang, N. Zhang, H. Shan, K. Su, Z. Meng, H. Kong and Z. Chen (2010). "Interactions among Proteins of Floral MADS-Box Genes in Basal Eudicots: Implications for Evolution of the Regulatory Network for Flower Development." Molecular Biology and Evolution **27**(7): 1598-1611.

Moon, Y. H., J. Y. Jung, H. G. Kang and G. H. An (1999). "Identification of a rice APETALA3 homologue by yeast two-hybrid screening." Plant Molecular Biology **40**(1): 167-177.

Pabon-Mora, N., B. Sharma, L. D. Holappa, E. M. Kramer and A. Litt (2013). "The Aquilegia FRUITFULL-like genes play key roles in leaf morphogenesis and inflorescence development." Plant J **74**(2): 197-212.

Pan, Z. J., Y. Y. Chen, J. S. Du, Y. Y. Chen, M. C. Chung, W. C. Tsai, C. N. Wang and H. H. Chen (2014). "Flower development of Phalaenopsis orchid involves functionally divergent SEPALLATA-like genes." New Phytol **202**(3): 1024-1042.

Pelaz, S., C. Gustafson-Brown, S. E. Kohalmi, W. L. Crosby and M. F. Yanofsky (2001). "APETALA1 and SEPALLATA3 interact to promote flower development." Plant Journal **26**(4): 385-394.

Rijpkema, A. S., J. Zethof, T. Gerats and M. Vandenbussche (2009). "The petunia AGL6 gene has a SEPALLATA-like function in floral patterning." Plant Journal **60**(1): 1-9.

Ruokolainen, S., Y. P. Ng, V. A. Albert, P. Elomaa and T. H. Teeri (2010). "Large scale interaction analysis predicts that the Gerbera hybrida floral E function is provided both by general and specialized proteins." BMC Plant Biol **10**: 129.

Seok, H. Y., H. Y. Park, J. I. Park, Y. M. Lee, S. Y. Lee, G. An and Y. H. Moon (2010). "Rice ternary MADS protein complexes containing class B MADS heterodimer." Biochem Biophys Res Commun **401**(4): 598-604.

Shchennikova, A. V. (2004). "Identification and Characterization of Four Chrysanthemum MADS-Box Genes, Belonging to the APETALA1/FRUITFULL and SEPALLATA3 Subfamilies." Plant Physiology **134**(4): 1632-1641.

Shitsukawa, N., C. Tahira, K.-i. Kassai, C. Hirabayashi, T. Shimizu, S. Takumi, K. Mochida, K. Kawaura, Y. Ogihara and K. Murai (2007). "Genetic and epigenetic alteration among three homoeologous genes of a class E MADS box gene in hexaploid wheat." Plant Cell **19**(6): 1723-1737.

Su, K., S. Zhao, H. Shan, H. Kong, W. Lu, G. Theissen, Z. Chen and Z. Meng (2008). "The MIK region rather than the C-terminal domain of AP3-like class B floral homeotic proteins determines functional specificity in the development and evolution of petals." New Phytologist **178**(3): 544-558.

Tsaftaris, A., K. Pasentsis, A. Makris, N. Darzentas, A. Polidoros, A. Kalivas and A. Argiriou (2011). "The study of the E-class SEPALLATA3-like MADS-box genes in wild-type and mutant flowers of cultivated saffron crocus (Crocus sativus L.) and its putative progenitors." J Plant Physiol **168**(14): 1675-1684.

Uimari, A., M. Kotilainen, P. Elomaa, D. Yu, V. A. Albert and T. H. Teeri (2004). "Integration of reproductive meristem fates by a SEPALLATA-like MADS-box gene." Proc Natl Acad Sci U S A **101**(44): 15817-15822.
