# Supplementary material for: Functional Conservation and Divergence of Four Ginger AP1/AGL9 MADS–Box Genes Revealed by Analysis of Their Expression and Protein–Protein Interaction, and Ectopic Expression of AhFUL Gene in Arabidopsis
Source: PLoS One. 2014 Dec 2;9(12):e114134. doi: 10.1371/journal.pone.0114134 (PMC4252096; doi:10.1371/journal.pone.0114134)
Supplement: Table S4 — Matrices for ancestral state reconstructions. Note: 0 for absence of interaction, 1 for presence of interaction and dash for no experimental record so far. (DOCX) [file pone.0114134.s009.docx]

**Table S4. Matrixes for ancestral state reconstructions**

|  | **AP1-AP1** | **AGL9-AGL9** | **AGL2-AGL2** | **AGL6-AGL6** | **AP1-AGL9** | **AP1-AGL2** | **AP1-AGL6** | **AGL2-AGL9** | **AGL6-AGL9** | **AGL2-AGL6** |
| --- | --- | --- | --- | --- | --- | --- | --- | --- | --- | --- |
| *Dendrathema grandiflorum* | 0 | --- | --- | --- | 1 | --- | --- | --- | --- | --- |
| *Nicotiana tabacum* | --- | --- | --- | --- | --- | 1 | --- | --- | --- | --- |
| *Solanum lycopersicum* | 0 | 0 | 1 | --- | 1 | 1 | --- | 0 | --- | --- |
| *Gerbera hybrida* | 1 | 1 | 1 | 0 | --- | --- | --- | 1 | 1 | --- |
| *Petunia hybrida* | 0 | 1 | 1 | 0 | 1 | 1 | --- | 1 | 1 | --- |
| *Antirrhinum majus* | 1 | 0 | --- | --- | 1 | --- | --- | --- | --- | --- |
| *Glycine max* | --- | --- | --- | --- | 1 | --- | --- | --- | --- | --- |
| *Arabidopsis thaliana* | 1 | 0 | 1 | --- | 1 | 1 | --- | 1 | 1 | --- |
| *Brassica oleracea var.botrytis* | 1 | --- | --- | --- | --- | --- | --- | --- | --- | --- |
| *Pachysandra terminalis* | 1 | 1 | --- | --- | 1 | --- | --- | --- | --- | --- |
| *Akebia trifoliata* | 1 | 1 | 1 | --- | 1 | 1 | --- | 1 | --- | --- |
| *Aquilegia coerulea* | --- | --- | --- | --- | 1 | 1 | 1 | --- | --- | --- |
| *Epimedium sagittatum* | 1 | --- | 1 | 1 | --- | --- | 0 | --- | --- | 1 |
| *Euptelea pleiospermum* | 0 | 1 | 0 | --- | 1 | 1 | --- | 1 | --- | --- |
| *Triticum aestivum* | --- | 1 | 1 | --- | 1 | 0 | --- | 1 | --- | --- |
| *Lolium perenne* | 1 | --- | --- | --- | --- | --- | --- | --- | --- | --- |
| *Oryza sativa* | 0 | 1 | 1 | --- | 1 | 1 | 1 | 1 | 1 | --- |
| *Phyllostachys praecox* | 1 | --- | --- | --- | --- | --- | --- | --- | --- | --- |
| *Alpinia hainanensis* | 0 | 1 | 0 | 0 | 1 | 1 | 1 | 1 | 1 | 1 |
| *Phalaenopsis equestris* | --- | 1 | 0 | --- | --- | --- | --- | --- | 1 | 1 |
| *Crocus sativus L.* | --- | --- | --- | --- | 1 | --- | --- | --- | --- | --- |
| *Chloranthus spicatus* | 0 | 1 | --- | --- | 1 | 1 | --- | --- | --- | --- |
