# Supplementary material for: Functional Conservation and Divergence of Four Ginger AP1/AGL9 MADS–Box Genes Revealed by Analysis of Their Expression and Protein–Protein Interaction, and Ectopic Expression of AhFUL Gene in Arabidopsis
Source: PLoS One. 2014 Dec 2;9(12):e114134. doi: 10.1371/journal.pone.0114134 (PMC4252096; doi:10.1371/journal.pone.0114134)
Supplement: Table S5 — Genes cloned in this study. (DOCX) [file pone.0114134.s010.docx]

**Table S5. Genes cloned in this study**

| gene name | lineage | amino acid | motif | localization |
| --- | --- | --- | --- | --- |
| *AhFUL* | *SQUA/FUL* | 244 | FUL, paleoAP1 | nucleus |
| *AhAGL6-like* | *AGL6* | 240 | AGL6-Ⅰ,AGL6-Ⅱ | nucleus |
| *AhSEP4* | *AGL2* | 242 | SEP-Ⅰ,SEP-Ⅱ | nucleus |
| *AhSEP3b* | *AGL9* | 215 | SEP-Ⅰ,SEP-Ⅱ | nucleus |
