# Supplementary material for: Functional Conservation and Divergence of Four Ginger AP1/AGL9 MADS–Box Genes Revealed by Analysis of Their Expression and Protein–Protein Interaction, and Ectopic Expression of AhFUL Gene in Arabidopsis
Source: PLoS One. 2014 Dec 2;9(12):e114134. doi: 10.1371/journal.pone.0114134 (PMC4252096; doi:10.1371/journal.pone.0114134)
Supplement: Table S6 — Expression patterns of AP1 / AGL9 genes comparison in Arabidopsis thaliana , Oryza sativa and Alpinia hainanensis . Different color represent different plant species, yellow, green and purple represent Arabidopsis thaliana, Oryza sativa and Alpinia hainanensis respectively. E: Genes with known expression in roots, leaves, inflorescence and floral meristems, bract, sepals, petals, stamens, carpels, and fruits are indicated. (DOCX) [file pone.0114134.s011.docx]

**Table S6. *AP1*/*AGL9* expression patterns comparison in *Arabidopsis*, *Oryza* and *A.hainanensis***

|  |  |  | root | leave | meristem | bract | sepal | petal | labellum | stamen | carpel | fruit |
| --- | --- | --- | --- | --- | --- | --- | --- | --- | --- | --- | --- | --- |
| *AP1/FUL* | *Arabidopsis* | *AP1* |  |  | E |  | E | E |  |  |  |  |
|  |  | *FUL* |  | E | E |  |  |  |  |  | EE | EE |
|  |  | *CAL* |  |  | E |  |  |  |  |  |  |  |
|  |  | *AGL79* | E | E | E |  |  |  |  |  |  | E |
|  | *Oryza* | *OsMADS14* |  |  | EE |  | EE |  |  | EE | E |  |
|  |  | *OsMADS15* |  |  | EE | E | E | E |  |  |  |  |
|  |  | *OsMADS18* | E | E |  |  | E | E |  | E | E |  |
|  | *Alpinia* | *AhFUL* |  | E |  | E | E |  |  |  | E |  |
| *AGL6* | *Arabidopsis* | *AGL6* | E | E | E |  | E | E |  |  | E |  |
|  |  | *AGL13* |  |  |  |  |  |  |  | E | E |  |
|  | *Oryza* | *OsMADS6* |  |  |  | E | E | E |  | E | E |  |
|  |  | *OsMADS17* |  |  |  | E | E | E |  | E | E |  |
|  | *Alpinia* | *AhAGL6-like* |  |  |  | E | E | E | E/- | E | E |  |
| *SEP1/2/4* | *Arabidopsis* | *SEP1* |  |  |  |  | E | E |  | E | E |  |
|  |  | *SEP2* |  |  |  |  | E | E |  | E | E |  |
|  |  | *SEP4* |  |  |  |  | E | E |  |  | E |  |
|  | *Oryza* | *OsMADS1* |  |  | E | E | E |  |  |  | E |  |
|  |  | *OsMADS5* |  |  |  |  |  |  |  | E | E |  |
|  |  | *OsMADS34* | E | E | E | E | E | E |  | E | E |  |
|  | *Alpinia* | *AhSEP4* |  | E |  | E | E | E | E | E | E |  |
| *SEP3* | *Arabidopsis* | *SEP3* |  |  |  |  |  | E |  | E | E |  |
|  | *Oryza* | *OsMAD7* |  |  | E |  |  | E |  | E | E |  |
|  |  | *OsMAD8* |  |  | E |  |  | E |  | E | E |  |
|  | *Alpinia* | *AhSEP3b* |  |  |  |  | E | E | E | E | E |  |
